# Cappable-Seq reveals the transcriptional landscape of stress responses in the bacterial endosymbiont Wolbachia

**DOI:** 10.1099/mgen.0.001542

**Published:** 2025-10-27

**Authors:** Youseuf Suliman, Zhiru Li, Amit Sinha, Philip D. Dyer, Catherine S. Hartley, Laurence Ettwiller, Alistair C. Darby, Clotilde K. Carlow, Benjamin L. Makepeace

**Affiliations:** 1 Institute of Infection, Veterinary & Ecological Sciences, University of Liverpool, Liverpool, Merseyside, L3 5RF, UK; 2 New England Biolabs, Ipswich, MA, 01938, USA

**Keywords:** cytoplasmic incompatibility, doxycycline, heat stress, operon, rifampicin

## Abstract

Bacterial endosymbionts are highly prevalent among invertebrate animals, in which they can confer fitness benefits such as pathogen defence and/or act as reproductive manipulators, inducing phenotypes including cytoplasmic incompatibility (CI). For the alpha-proteobacterium *Wolbachia*, its wide distribution among macroparasites and disease-transmitting arthropods coupled with mutualistic roles, reduction of vector competence and CI has found recent applications in the control of several vector-borne tropical diseases. However, in common with other bacterial endosymbionts, which often lose regulatory elements during genomic erosion, the degree to which *Wolbachia* can respond to environmental or pharmacological stressors is poorly understood. Here, we apply Cappable-Seq methodology to achieve unprecedented depth and resolution of transcriptional start sites (TSS) in two *Wolbachia* strains (*w*MelPop-CLA and *w*AlbB) that have been used to transinfect mosquitoes for arbovirus control. We exposed *Wolbachia* in mosquito cell lines to temperature stress (both strains) or antibiotics (*w*AlbB only) and observed that all classes of TSS (including antisense) exhibited differential regulation, some of which were associated with mobile elements and may control ncRNA expression. Of the three antibiotics used as pharmacological stressors (doxycycline, rifampicin and moxifloxacin), doxycycline had the greatest impact on differential expression from primary TSS. Cappable-Seq also resolved the organization of the bicistronic *cifA/cifB* operon that is responsible for inducing CI in *Wolbachia* hosts. The use of Cappable-Seq in this study enabled the resolution of the primary transcriptome of an obligate intracellular bacterium in unparalleled detail. Moreover, this methodology shows great promise for revealing regulation of symbiont functions in whole invertebrates.

**Impact Statement**

*Wolbachia* is the most prevalent endobacterial symbiont of invertebrate animals worldwide, infecting most terrestrial arthropods and some nematodes. *Wolbachia* has profound ecological and evolutionary impacts on its hosts via both beneficial and detrimental effects, including reproductive parasitism, nutrient provisioning, and protection against pathogens; it also maintains obligate relationships with some filarial nematodes and a few arthropods. These characteristics have led *Wolbachia* to be either deliberated introduced into mosquito populations to reduce arboviral transmission or targeted by antibiotic therapy to cure filarial diseases. Despite these important applications of *Wolbachia* in disease control, many aspects of its biology, such as its ability to respond to stressors, remain enigmatic due to genetic intractability. Here, we applied a methodology termed Cappable-Seq to dissect genome-wide regulation of gene expression in *Wolbachia* for the first time while applying two types of stressors: temperature and antibiotics. Despite its reduced genome, *Wolbachia* exhibited complex patterns of differential expression across all types of transcriptional start sites in response to stress, with evidence that ncRNAs may compensate for the loss of protein-coding regulatory genes during genome decay. These findings have implications not only for reductive genome evolution in symbionts, but also for the behaviour of *Wolbachia* in disease control contexts.

## Data Availability

Raw data are available at the SRA database under accession no. PRJNA1145481.

## Introduction

Symbiosis, the co-occurrence of dissimilar organisms in a long-term interrelationship, encompasses a highly diverse range of interactions involving every kingdom of life. Bacteria are particularly pervasive symbionts of eukaryotes and, in many cases, have evolved to become completely dependent on their hosts for nutrition and propagation, replicating intracellularly in an obligate dependency. In the most highly specialized symbioses, vertical transmission of the endosymbiont becomes the primary or sole mode of transfer to a new host, usually via the maternal line only [1]. As a consequence of relaxed selection and the population bottlenecks enforced by vertical transmission, genome streamlining is a key feature of inherited endosymbionts, and this is accompanied by an accumulation of deleterious mutations (the so-called ‘Muller’s ratchet’ [1]). In the most extreme cases, the bacterial genome becomes vestigial, encoding only a minimized suite of genes to support a mutualistic relationship with the host, and the distinction between endosymbiont and organelle becomes blurred [2]. However, streamlining can be slowed or even reversed by expansions of mobile elements in endosymbiont genomes; indeed, some symbiotic bacteria have higher proportions of genomic content comprised of prophages and transposons than free-living species do [3,4].

A common consequence of population bottlenecks in inherited endosymbionts is a loss of DNA repair and recombination genes, which fuels the further erosion of coding capacity through pseudogenization [2]. Consequently, the ability of an endosymbiont to regulate gene expression becomes hampered by the depletion of sigma factors and other regulatory components [5]. The gamma-proteobacterial symbiont of pea aphids, *Buchnera aphidicola*, which has a genome size of 641 kb, is a model of genome reduction in a strictly vertically transmitted mutualist. This organism has retained only two sigma factor genes, *rpoD* (encoding housekeeping σ^70^) and *rpoH* (encoding heat-shock σ^32^), and eight transcriptional regulators [6]. In experiments designed to probe the ability of *B. aphidicola* to respond at the transcriptomic level to heat stress or changes in nutritional demand due to host diet restriction, limited evidence for regulatory capacity was apparent [7]. However, *B. aphidicola* did regulate its transcriptome in response to host genetic background, which influences symbiont titre [8]; although within isoclonal aphid lines, *B. aphidicola* density was affected by host rearing temperature without differential expression (DE) in the endosymbiont [9]. Importantly, at the protein level, differential regulation was identified in *B. aphidicola* between anatomical compartments of aphids in the absence of corresponding changes in mRNA expression [6]. These protein-level differences were hypothesized to be controlled post-transcriptionally by intergenic and antisense small RNAs (sRNAs); moreover, some sRNAs were found to be differentially expressed in *B. aphidicola* when the aphid host was fed on different plants [10].

Unlike *Buchnera*, the alpha-proteobacterium *Wolbachia* has a vast host range in arthropods and some parasitic nematodes, representing the most prevalent symbiont of animals on the planet [4]. *Wolbachia* is of major applied significance due to its proven efficacy in controlling pest insects or reducing their ability to spread disease [11], as well as representing a drug target for the treatment of filarial nematodes that cause two neglected tropical diseases: lymphatic filariasis and onchocerciasis [12]. Whereas *Wolbachia* in nematodes (and rare mutualistic *Wolbachia* strains in arthropods) have very small genomes in a similar range to *Buchnera* (0.5–1 Mb) [13,15], most *Wolbachia* strains are facultative reproductive parasites of arthropods and have larger genomes (1.2–2.2 Mb) [16,17]. These contain mobile elements such as prophages, transposons and plasmids, and recombination between *Wolbachia* strains with facultative lifestyles is possible due to uncommon but evolutionarily significant horizontal transmission events [18]. Of the few transcriptomic studies performed on *Wolbachia*, the majority have examined DE across the lifecycle of the host or between host tissues in filariae, with a recent meta-analysis concluding that *Wolbachia* exhibits only a weak regulatory response involving upregulation of ribosomal proteins during larval development of the host [19]. One study analysed the transcriptomic and proteomic response of a *Wolbachia* strain from *Drosophila melanogaster*, *w*MelPop-CLA, to doxycycline treatment in an insect cell line system [20]. Although only 5% of genes were differentially regulated during treatment, the tolerance of *Wolbachia* to short-term therapy with tetracyclines demonstrates that this plasticity is biologically relevant, enabling a proportion of bacterial cells to survive a potent stressor. *Wolbachia* is also capable of DE of certain genes between host sexes [21]; moreover, during the host lifecycle, it actively migrates from somatic to germline tissues [22,23]. These observations suggest that it responds to cues in the intracellular environment of the host to enable temporally restricted phenotypic changes. Similarly, manipulation of the host diet in *Drosophila* affects *Wolbachia* density between somatic and germline compartments, leading to alterations in symbiont nucleoid morphology [24], although transcriptional changes in *Wolbachia* in response to host diet have not been explored.

Despite major technical advances in sequencing technology over the past 15 years, transcriptomic studies of endosymbionts that cannot be cultured axenically remain challenging. This is because the abundance, and therefore sequence coverage, of host transcripts and bacterial rRNA is several orders of magnitude greater than that of endosymbiont mRNA. There are three main approaches to overcoming this problem. First, host and bacterial rRNA can be depleted using probes complementary to these hyperabundant transcripts [25]. This technique relies either on the use of commercial kits, where the match to the rRNA species in the samples may not be optimal, or the synthesis of custom probe sets. However, even if probes have high complementarity to the target, they are limited in the scale of depletion that can be achieved. Second, terminator 5′-phosphate-dependent exonuclease (TEX) can be used to digest 5′-phosphate-associated rRNA [26], although this method still lacks optimum efficacy, as the enzymatic degradation is incomplete. Finally, bait libraries, such as the Agilent SureSelect system, can be used with predesigned probes to capture rare transcripts within a mixed RNA pool [27]. Although the Agilent SureSelect system is highly effective at enriching mappable reads, probe design can omit unique genes (e.g. sRNAs) that may be absent in genome annotations. It is also possible that probes could capture degraded RNA; thus, the enriched transcripts may not accurately represent the nascent transcriptional activity of the endosymbiont.

Recently, a method has been developed which involves the selective capping of prokaryotic 5′-triphosphate (5′PPP) RNA with biotinylated guanosine triphosphate (GTP), effectively separating primary prokaryotic mRNA from host mRNA and bacterial rRNA in the RNA pool. This method is termed Cappable-Seq [28] and has been successfully applied to the *Wolbachia* symbiont of the filarial worm, *Brugia malayi* [29]. This study demonstrated up to fivefold enrichment of non-rRNA transcripts within *Wolbachia*, allowing a more comprehensive transcriptional profiling of the endosymbiont over conventional methods. A benefit of the selective enrichment of 5′PPP is that it can define the transcriptional start site (TSS) of the captured nascent primary mRNA at single-nucleotide resolution, highlighting an early transcriptional snapshot of gene expression within the bacterium without the need for predesigned probes.

Here, using stable infections in mosquito cell lines, we apply Cappable-Seq to two *Wolbachia* endosymbionts from insects: *w*MelPop-CLA from *D. melanogaster* [30] and *w*AlbB from the tiger mosquito, *Aedes albopictus* [31]. Both strains have been used in mosquito control and cause cytoplasmic incompatibility (CI), in which crosses between uninfected male hosts with infected females, or males and females infected with incompatible *Wolbachia* strains, lead to embryonic lethality [4]. In the context of mosquito control, CI has been used either for (a) population suppression, in which releases of incompatible males cause wild populations to crash, or (b) population replacement, in which *Wolbachia*-free wild populations are supplanted by *Wolbachia*-transinfected individuals (males and females) because the symbiont interferes with arbovirus transmission [32]. An additional application of *w*AlbB has been as a surrogate for endosymbionts of filarial worms (which cannot be cultured) in antibiotic screening programmes using *Wolbachia*-infected insect cell lines [33].

In this most extensive TSS study performed in an obligate intracellular bacterium to date, we discover various canonical or primary TSS (pTSS) in the region immediately upstream of the corresponding gene, as well as multiple non-canonical TSS subtypes, comprising secondary, gene-associated TSS (gTSS), intragenic TSS (iTSS), antisense TSS (asTSS) and orphan TSS (oTSS) – as has been reported in other bacteria [26,34,37]. We uncover pervasive non-canonical TSS usage and DE in both strains, including a high proportion of asTSS, some of which are associated with putative sRNAs. The two strains show divergent transcriptional profiles under temperature stress and differ greatly in expression of TSS linked to mobile elements (insertion sequences and prophages). Finally, in strain *w*AlbB, we reveal distinct transcriptional responses to different classes of antibiotics that have been proposed for use for filarial disease control.

## Methods

### Cell culture

To investigate the transcriptional response of *w*MelPop-CLA and *w*AlbB exposed to stress, mosquito cell lines were used as a model to study *Wolbachia* gene expression within the background of a host relevant to current vector control efforts. The RML-12 and Aa23 *Ae. albopictus* cell lines harbouring *w*MelPop-CLA [30] and *w*AlbB [31], respectively, were maintained at 28 °C in T75 flasks. Cell lines were grown in 15 ml insect media composed of 45% Mitsuhashi and Maramorosch medium (HiMedia), 45% Schneider’s *Drosophila* medium (Sigma), 10% FCS and 0.18% l-glutamine. Cell lines were passaged every 4–6 days until confluency in a 1 : 5 ratio (3 ml suspended cells plus 12 ml fresh media).

### Temperature stress induction

For temperature stress, mosquito cells were maintained in either T25 (RML-12: 5 ml cultures) or T225 (Aa23: 45 ml cultures) flasks at 28 °C for 5–7 days and then transferred to incubators at each temperature (21 °C, 28 °C and 34 °C) for either 4, 24 or 48 h before harvest, with 3 biological replicates per condition. Temperatures were selected to reflect values experienced in tropical climates such as the wet season in Cairns, Australia [38], whilst avoiding temperature limits that may damage the host cell line during continuous exposure [39]. Once the incubation periods were complete, cells were prepared for RNA extraction.

Due to the presence of low-density *w*MelPop-CLA, RML-12 sample preparation differed from Aa23 as follows. The RML-12 cells were processed by resuspension and transfer into 50 ml Falcon tubes placed directly onto ice to stall metabolic and transcriptional activity. Five millilitres of sterile 3 mm glass beads were added to the cell suspension and vortexed for 5 min to lyse mosquito cells and liberate intracellular *Wolbachia*. The vortexed cell suspension was then centrifuged at 2,500 ***g*** at 4 °C for 15 min to pellet host cell debris. The *Wolbachia*-containing supernatant was subsequently filtered through a 5 µm syringe filter, then further centrifuged at 18,000 ***g*** at 4 °C for 15 min to pellet *Wolbachia* cells. The supernatant was removed, and the *Wolbachia* pellet was resuspended in 1 ml media and further filtered through a 2.7 µm syringe filter into a 1.5 ml Eppendorf tube. After a final centrifugation step at 18,000 ***g*** at 4 °C for 15 min, the *Wolbachia* pellets were suspended in 1 ml QIAzol and stored at −80 °C. For Aa23, cells were resuspended and transferred into 50 ml Falcon tubes and centrifuged at 300 ***g*** at 4 °C for 10 min. Aa23 cells were also resuspended in and transferred to 50 ml Falcon tubes but did not undergo cell bead lysis; instead, the cell suspension was centrifuged at 2,500 ***g*** at 4 °C for 15 min followed by removal of the supernatant. The remaining cell pellet was then suspended in 1 ml QIAzol and stored at −80 °C.

### Antibiotic stress induction

The *w*AlbB antibiotic stress induction was performed by growing Aa23 cells in T25 flasks (5 ml culture, triplicate) for 6 days. Antibiotics were added at 0.25 µg ml^−1^ (doxycycline), 0.25 µg ml^−1^ (rifampicin) and 1 µg ml^−1^ (moxifloxacin) for 24 h. Cells were then harvested by resuspending cells and transferring them into 15 ml Falcon tubes. Samples were centrifuged at 300 ***g*** for 5 min, the supernatant removed and 2 ml of QIAzol added to each cell pellet prior to storage at −80 °C. Antibiotic dosages were based on physiologically relevant concentrations and those chosen by previous studies [20,40, 41].

### RNA extraction

RNA was extracted via QIAzol-chloroform extraction. Briefly, 200 µl of 100% chloroform was added per millilitre of QIAzol-suspended sample and shaken by hand for 15 s. Samples were incubated at room temperature for 3 min and then centrifuged at 12,000 ***g*** at 4 °C for 15 min. After incubation, 500 µl of the aqueous phase was transferred to DNA LoBind tubes (Eppendorf) to which 750 µl of absolute ethanol was added. Total RNA was extracted from each sample using the Monarch total RNA miniprep kit [New England Biolabs (NEB) product number T2010S] TRIzol extraction protocol with on-column DNase treatment, as specified by the manufacturer. The RNA concentrations were assessed using a NanoDrop spectrophotometer (Thermo Fisher Scientific).

### Cappable-Seq enrichment

For the capping reaction, 30 µl of 2–10 µg of DNase-treated RNA was added to 5 µl of 10× vaccinia capping enzyme buffer (NEB product number M2080) with 5 µl of 5 mM 3′-desthiobiotin-guanosine triphosphate (DTB-GTP) (NEB product number N0761), 5 µl vaccinia capping enzyme (NEB product number M2080), 5 µl inorganic pyrophosphate (NEB product number M2403) and ultrapure water (as needed) to a total volume of 50 µl and incubated at 37 °C for 1 h. Samples underwent RNA purification using the Monarch RNA clean-up kit (NEB product number T2030S), modified to increase the washing step to a total of five times to ensure removal of free DTB-GTP; RNA was then transferred to a PCR tube. The DTB-GTP capped RNA underwent fragmentation by adding 1.25 µl of 10X T4 polynucleotide kinase buffer and was incubated at 94 °C for 5 min, then placed directly on ice. The removal of the 3′ phosphates from fragmented RNA was accomplished by adding 7 µl of 10X T4 polynucleotide buffer and 3.2 µl of ATP-free T4 polynucleotide kinase, followed by incubation at 37 °C for 15 min. Samples then underwent RNA purification using the Monarch RNA clean-up kit (NEB T2030S) and were eluted with 40 µl ultrapure water.

Samples were subjected to two rounds of enrichment with streptavidin beads by adding 30 µl of DTB-GTP-capped RNA to 30 µl of preprepared streptavidin beads and placed onto a rotation mixer at room temperature for 30 min. Samples were then placed onto a magnetic stand for 5 min or until the solution was clear. Whilst on the magnetic stand, samples were washed four times with 200 µl washing buffer (10 mM Tris-HCl, pH 7.5, 120 mM NaCl, 1 mM EDTA). Beads were resuspended in 11 µl low TE elution buffer and incubated in a thermomixer at 80 °C for 10 min before a magnetic stand was used to facilitate recovery of RNA. Bead resuspension was performed twice before a second round of streptavidin enrichment was performed with 20 µl of DTB-GTP-capped RNA and 20 µl of streptavidin beads. Decapping of the 5′ DTB-GTP cap was performed to leave the 5′ monophosphate for ligation by adding 20 µl of capped RNA to 2.2 µl of 10X ThermoPol buffer (NEB product number B9004) and 2 µl RppH (NEB product number M0356S), followed by incubation at 37 °C for 1 h. Next, 1 µl of 1 : 10 diluted proteinase K was added to the solution and incubated at 37 °C for 10 min. The RNA solution then underwent a final round of RNA purification using the Monarch RNA clean-up kit (NEB T2030S) before library preparation.

### Library preparation and sequencing

Enriched RNA underwent library preparation using the NEBNext multiplex small RNA library prep kit (NEB product number E7300S) following the manufacturer’s instructions. After library completion, samples underwent PCR clean-up by adding 1 vol of Ampure beads followed by incubation at room temperature for 10 min. Samples were placed onto a magnetic stand until the solution became clear. The supernatant was removed, and beads were washed with 80% ethanol four times, air-dried, resuspended in 40 µl low TE buffer and incubated at room temperature for 10 min. The supernatant was collected, and then the resulting library was diluted 1 : 10 and analysed using the high-sensitivity DNA Agilent Bioanalyzer protocol using a DNA 1000 chip according to the manufacturer’s instructions. Samples were sequenced on an Illumina NEXTSeq using high-output single-end 150 bp sequencing.

### Mapping

Adapter trimming and contaminant filtering were performed using BBduk from the BBtools software package (version 38.86). Unpaired single-end reads were mapped using Bowtie2 (version 2.4.5) with the local alignment option. Reads were mapped simultaneously onto a concatenated FASTA file containing the genomes of *Ae. albopictus* mitochondria (accession NC_006817), *Ae. albopictus* (assembly GCA_001876365.2) and *w*Mel (assembly GCA_000008025.1) or *w*AlbB (assembly GCA_004171285.1). A threshold for a minimal sequencing depth (Fig. S1 Supplementary Material 1, available in the online Supplementary Material) was chosen based on the recommended minimal sequencing depth of two million for differential gene expression in *E. coli* [42]. The two million *E. coli* minimal read depth equates to 0.43 million reads per Mb of genome length, or a minimal desired library depth of 0.54 M and 0.63 M reads for *w*MelPop-CLA and *w*AlbB, respectively. The *w*MelPop-CLA genome is highly similar to *w*Mel, besides defined minor differences [43], allowing *w*Mel to be a suitable proxy for gene expression in *w*MelPop-CLA. Aligned reads that mapped to *Wolbachia* were further filtered for those with a unique mapping score of mapq20 using samtools (version 1.11) to ensure reads associated with repetitive regions were confidently assigned. Removing non-uniquely mapped reads may limit additional information from repeat elements, such as transposons; however, their removal provided greater confidence in the expression of TSS that remained associated with repeat elements. Reads were then converted into count values using the software HTSeq-count (version 0.11.1).

### TSS calling

The TSS calling of mapped reads was conducted using the TSS calling software pipeline (accessible from https://github.com/Ettwiller/TSS) using a clustering filter of a ten-nucleotide window. Designating a TSS type was performed using a custom Python script implementing pandas and Bedtools closest (v2.29.2). The TSS types were assigned based on the location of the TSS relative to the closest neighbouring genes using the following criteria: pTSS had the highest read count within 300 nucleotides upstream of the start codon; gTSS were within 300 nucleotides upstream to the start codon, but with read counts less than the pTSS; iTSS were internal to the CDS; asTSS were antisense to CDS and/or 100 nucleotides downstream to the antisense stop codon; and oTSS comprised those TSS not fulfilling any of the previous definitions. High-confidence TSS were determined by only selecting those TSS which were present in all replicates within a condition. Any TSS that were absent in a condition but present in others were given a prior count of 10. To apply the same minimal expression value to all samples, the threshold for a TSS to be called was 10 c.p.m., based on the smallest sample library for temperature and antibiotic experiments separately. The TSS statistics and graphs were generated via the Python library packages matplotlib and seaborn. Heatmaps were generated using heatmap.2 in the R package ggplot2 (version 3.3.2).

### 5′ RACE analysis

To confirm the single-nucleotide accuracy of Cappable-Seq, 5′ RACE analysis was conducted on a highly expressed gene (*rpsM*) encoding the uS13 protein of the 30S ribosomal subunit of *w*AlbB (Table S1, Supplementary Material 1). The study used the NEB 5′ RACE protocol using the template switching RT enzyme mix (NEB product number M0466) and cloned into a single-use stable competent *E. coli* using the NEB PCR cloning kit (NEB product number E1203S) according to the manufacturer’s instructions.

### Orthologous gene comparisons

To define orthologous genes between *w*Mel and *w*AlbB, the AA sequences of all protein-coding genes in each genome were compared via the OrthoFinder package (version 2.5.4) [44]. As a conservative approach to increase the likelihood of functional similarity between protein-coding genes, only those genes with protein products that shared one-to-one homology between *w*Mel and *w*AlbB were considered for further analysis.

### Motif discovery

To find sequence motifs indicative of promoter regions, the upstream 50-nucleotide regions of all TSS were used as input into the web browser-based MEME suite (version 5.4.1) with up to 10 motif patterns selected in the settings [45]. Motifs were visualized using the software package WebLogo (version 2.8.2) [46].

### DE analysis

DE analysis was conducted using the empirical analysis of digital gene expression package edgeR (version 3.30.3) [47] with the following settings: prior count of two, minimal fold change threshold of two and an adjusted *P*-value false discovery rate (Benjamini–Hochberg procedure) of 5%. Multidimensional scaling (MDS) plots were analysed and produced via the plotMDS function within edgeR.

### Functional annotation

To aid in the biological interpretation of DE TSS under stress, genes were assigned KEGG (Kyoto Encyclopedia of Genes and Genomes) Ortholog (KO) annotations, so they could be included in the KEGG pathway analysis. Functional gene annotations were retrieved with the use of EggNOG (version 5.0.0) [48]; KO annotations of DE genes were then used as input into the KEGG pathway analysis under organism prefixes of ‘wol’ and ‘wpp’ for *w*Mel and *w*AlbB, respectively. Pathways which resulted in the greatest number of hits or were associated with highly DE TSS were the focus of biological interpretation.

### RNA secondary structure analysis

To evaluate the likelihood of whether a TSS could be associated with an ncRNA instead of, or associated with, a protein-coding gene annotation, the 100 nucleotide downstream sequences of all TSS types were analysed for predicted RNA secondary structures with minimum free energy (MFE) values in RNAfold (version 2.4.13) [49] of the ViennaRNA package (version 2.0) using default settings. A total of 2,000 (1,000 per strand) randomly generated TSS locations were created with a custom python3 script as a comparative control.

### Operon structure analysis

To assess the structure of predicted operons within *w*MelPop-CLA and *w*AlbB, gene annotations for both strains were used as input for the Rockhopper tool (version 1.7.0.11) [50]. The BAM alignment files from 28 °C at 48 h conditions were used as input into Rockhopper to decipher whether the intergenic gaps between genes displayed evidence of co-expression. Operons and associated TSS were visualized using the Artemis genome browser and annotation tool (version 18.1.0). Expression of an operon was positively defined if the initiating gene of a predicted operon harboured a pTSS. Operons were considered to contain an alternative transcriptional unit (ATU) if they displayed a moderately expressed iTSS upstream to a gene within the associated operon.

## Results

### Enrichment of *Wolbachia* primary transcripts

We applied Cappable-Seq to enrich for primary *Wolbachia* transcripts in strains *w*MelPop-CLA and *w*AlbB and assessed the efficacy of the process (Table 1). Processed tRNA and rRNA constituted most *Wolbachia* transcripts in unenriched samples, representing 0.21 and 8.28% of the total RNA pool, respectively, or 88.454 and 89.807% of *Wolbachia*-specific transcripts for *w*MelPop-CLA and *w*AlbB, respectively. Following enrichment, the proportion of *Wolbachia* primary transcripts increased over 94-fold (0.027%–2.517%) and over 14-fold (0.939%–13.535%) for *w*MelPop-CLA and *w*AlbB, respectively (Table 1). However, reads mapping to host mitochondria were also enriched 55.494- and 2.481-fold for *w*MelPop-CLA and *w*AlbB, respectively, which is expected due to the prokaryotic origin of mitochondria.

**Table 1.** Mapping rates of reads (%) to various RNA categories without (unenriched) and with (enriched) Cappable-Seq

	*w*MelPop-CLA			*w*AlbB		
**RNA source**	**Unenriched**	**Enriched**	**Fold change**	**Unenriched**	**Enriched**	**Fold change**
**Host**	99.584	87.144	0.875	90.736	85.234	0.939
**Mitochondria**	0.185	10.241	55.494	0.048	0.119	2.481
***Wolbachia*** **(total)**	0.232	2.615	11.289	9.216	14.647	1.589
***Wolbachia*** **tRNA**	0.057	0.068	1.206	3.043	0.194	0.064
***Wolbachia*** **rRNA**	0.148	0.029	0.196	5.234	0.919	0.176
***Wolbachia*** **primary transcripts**	0.027	2.517	94.134	0.939	13.535	14.409

### Identification and categorization of *Wolbachia* transcriptional start sites

**Fig. 1.**
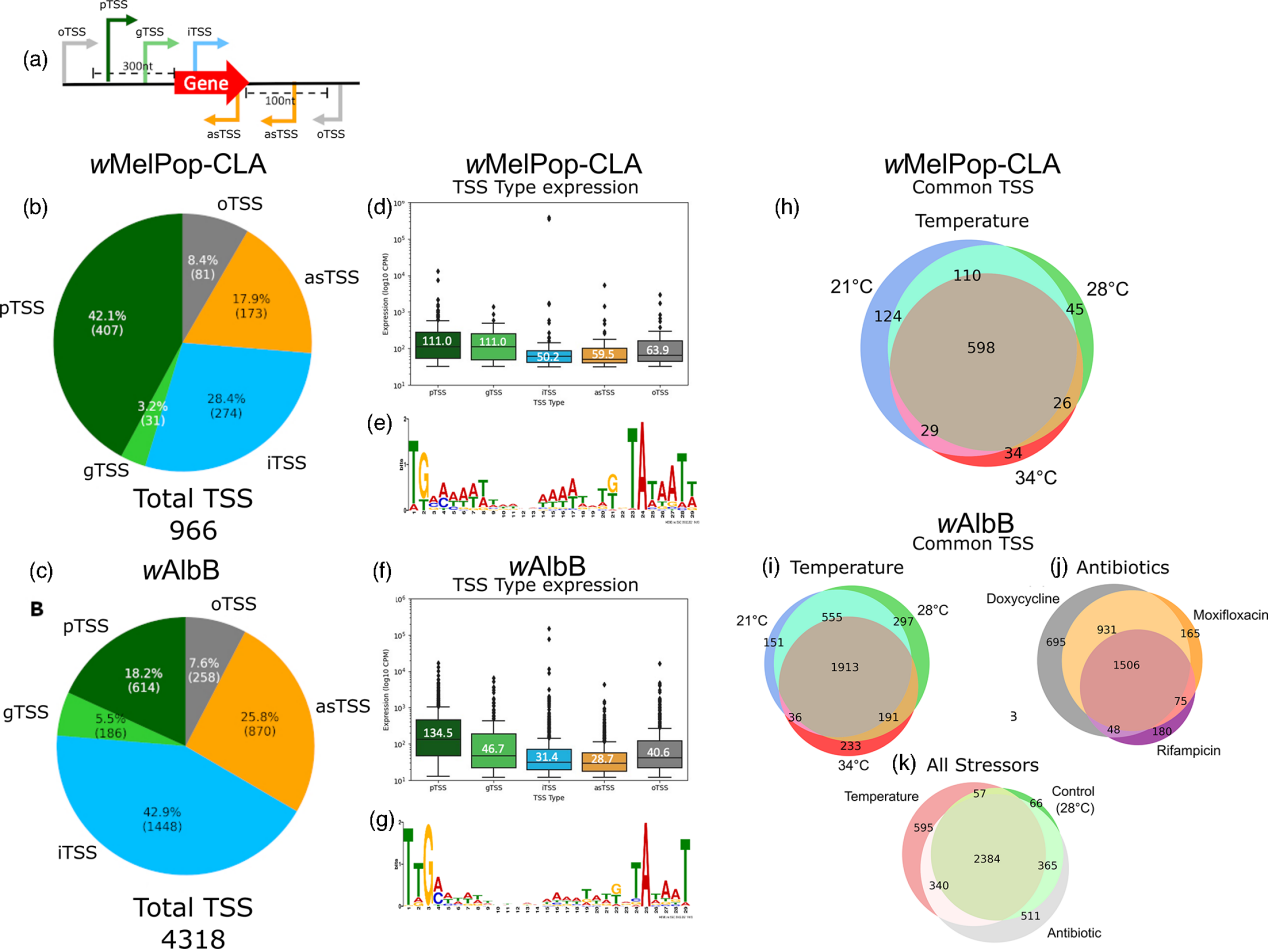
Overview of TSS characteristics for *w*MelPop-CLA and *w*AlbB. (a) Classification scheme for TSS types used in the present study. Types comprise ‘pTSS’, primary gene-associated TSS; ‘gTSS’, secondary gene-associated TSS; ‘iTSS’, sense internal to an annotated gene; ‘asTSS’, antisense to an annotated gene or within 100 nucleotides upstream antisense to a coding region; ‘oTSS’, oTSS without an associated gene. (b) Proportion of TSS types found in *w*MelPop-CLA. (c) Proportion of TSS types found in *w*AlbB. (d) TSS expression by type [median expression (log_10_ c.p.m.)] and (e) motif from the 100 nt upstream sequence of total TSS for *w*MelPop-CLA. (f) TSS expression by type [median expression (log_10_ c.p.m.)] and (g) motif from the 100 nt upstream sequence of total TSS for *w*AlbB. (h) Commonality of TSS between temperature stress conditions for *w*MelPop-CLA. (i) Commonality of TSS between temperature conditions for *w*AlbB. (j) Commonality of TSS between antibiotics for *w*AlbB. (k) Commonality of TSS between all stress conditions for *w*AlbB.

**Fig. 2.**
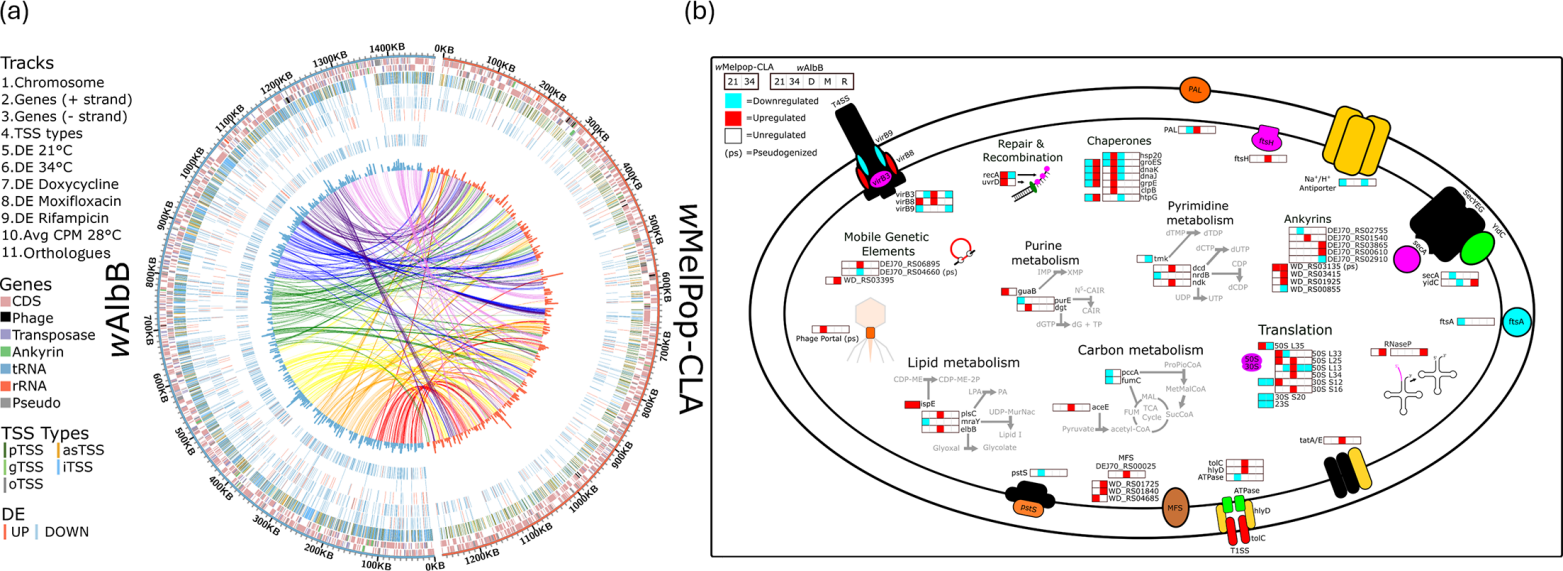
(a) Genome-wide comparative mapping of gene categories, TSS distribution, DE genes and orthologues between *w*AlbB and *w*MelPop-CLA. (b) Summary of DE pTSS and associated functional groups for both *w*MelPop-CLA and *w*AlbB under antibiotic and/or temperature stress. Metabolic pathways associated with enzymes are coloured grey. Protein structures present but not observed to be DE are filled in black. Stress conditions: 21=21 °C, 34=34 °C, D=doxycycline, R=rifampicin and M=moxifloxacin.

We mapped 24,872,374 and 151,846,942 unique single-end 150 bp reads representing primary *Wolbachia* transcripts to their TSS sites in *w*MelPop-CLA and *w*AlbB, respectively, and further classified each of the TSS sites to a specific subtype using the following criteria (Fig. 1a). The canonical pTSS is defined as a TSS site that occurs within 300 nucleotides upstream of the closest gene on the same strand. In cases where additional TSS sites with lower read counts were observed within this region, they were classified as secondary gTSS. The TSS peaks observed within a CDS were classified as iTSS if they were on the same strand as the gene, and as asTSS if they were on the opposite strand. The TSS sites that could not be placed in any of these categories were classified as oTSS. This resolved 966 and 4,318 TSS for *w*MelPop-CLA and *w*AlbB, respectively, among all conditions tested (Fig. 1b, c). The most abundant TSS type for *w*MelPop-CLA was pTSS (42.1%), whereas for *w*AlbB, iTSS were predominant (45.6%). Thus, a considerable discrepancy was apparent in the total TSS detected between *w*MelPop-CLA (0.76 per kb) and *w*AlbB (2.9 per kb). However, after matching sequencing depths and TSS thresholds, the total for *w*AlbB was reduced to 0.94 per kb (Fig. S2, Supplementary Material 1), mainly due to fewer iTSS. The least abundant TSS type for *w*MelPop-CLA and *w*AlbB was gTSS (3.2 and 5.4%, respectively). For the annotated genes of *w*MelPop-CLA and *w*AlbB (1,286 and 1,418, respectively), ~29.63% (407) and 40.62% (682) harboured a canonical pTSS, respectively. Strain *w*AlbB contained a greater proportion of intragenic TSS (iTSS and asTSS), with over half of all CDSs containing an iTSS (53.74%), and over a third containing an asTSS (39.56%), compared to *w*MelPop-CLA (iTSS, 16.10%; asTSS, 10.19%) (Fig. 2a). To confirm the single-nucleotide resolution of TSS assignment, we performed template-switching 5′ rapid amplification of cDNA ends (RACE) targeting the pTSS of a highly expressed 30S ribosomal subunit protein (uS13) gene, *rpsM*, of *w*AlbB. Cloned transcripts indicated both the dominant pTSS detected by Cappable-Seq and a secondary gTSS differing from the primary site by one nucleotide (Fig. S3, Supplementary Material 1).

The number of TSS expressed per condition ranged from 687 to 861 and 2,373–2,956 under temperature stress for *w*MelPop-CLA and *w*AlbB, respectively, and 1,809–3,180 for *w*AlbB under antibiotic stress, with excellent concordance between biological replicates (Fig. S4, Supplementary Material 1). Approximately one-half to two-thirds of expressed TSS were shared between temperature conditions for *w*MelPop-CLA (62%, *n*=598) and *w*AlbB (57%, *n*=1,913) (Fig. 1h–i); this was also apparent for *w*AlbB exposed to antibiotic stress (42%, *n*=1506, Fig. 1j). Conversely, for both *w*MelPop-CLA and *w*AlbB, a small proportion of TSS was expressed under specific conditions at 21 °C (13%, *n*=124 and 5%, *n*=151), 28 °C (5%, *n*=45 and 9%, *n*=297) and 34 °C (4%, *n*=34 and 7%, *n*=233), respectively (Fig. 1h–i). Condition-specific TSS were also exhibited by *w*AlbB between the three antibiotic treatments [doxycycline (19%, *n*=695), rifampicin (5%, *n*=180) and moxifloxacin (5%, *n*=165)]. Moreover, in *w*AlbB, a similar proportion of TSS specific for either temperature stress (14%, *n*=595) or antibiotic stress (12%, *n*=511) was apparent (Fig. 1k).

At baseline conditions (28 °C), pTSS was the most highly expressed TSS type in both *w*MelPop-CLA and *w*AlbB, with a median expression of 111.0 and 134.5 c.p.m., respectively (Fig. 1d, f). Although iTSS were highly abundant across the *Wolbachia* genomes, the median expression level for iTSS and asTSS was ranked as the lowest TSS type for both *w*MelPop-CLA and *w*AlbB. We applied motif-based sequence analysis to identify promoters in the 100 bp region upstream to each TSS site, and two distinct motifs were recognized: the common AT-rich −10 TATAAT box and a less frequent −35 TTGACA core promoter consensus sequence for σ^70^ (Fig. 1e, g). Analysis of motifs within the various subtypes of TSS also identified these promoter motifs in pTSS, iTSS and oTSS in both *w*Mel and *w*AlbB, while other motifs such as TAAAAT were found for gTSS and asTSS (Fig. S4).

Among the top 20 most highly expressed TSS, most TSS types were represented (excluding gTSS); 60% consisted of pTSS for both *w*MelPop-CLA and *w*AlbB (Table 2). The two most highly expressed TSS for both *w*MelPop-CLA and *w*AlbB were iTSS associated with the non-coding 6S RNA gene (*ssrS*) and the fructose-6-phosphate aldolase (*fsaA*) CDS. Using a single baseline sample (28 °C) for *w*MelPop-CLA and *w*AlbB, 38.3 and 8.7% of total reads were assigned to the *ssrS* iTSS (Fig. S6, Supplementary Material 1), and 33.9 and 8.7% to the *fsaA* iTSS (Fig. S6, Supplementary Material 1), respectively. Hypothetical proteins were also highly expressed in both strains, comprising substantial proportions of the top 20 TSS in *w*MelPop-CLA (5) and 30% in *w*AlbB (6). Notably, a pTSS for a phage tail protein (WD_RS01240) was highly abundant in the *w*MelPop-CLA dataset (Fig. S6, Supplementary Material 1), as was an asTSS for a major capsid protein (WD_RS01225; Fig. S6, Supplementary Material 1). Moreover, an asTSS associated with the mismatch repair protein gene *mutL* was present within the top 10 most highly expressed TSS for both *w*MelPop-CLA and *w*AlbB (Fig. S6, Supplementary Material 1).

**Table 2.** Top 20 expressed TSS at baseline (28 °C). Mean c.p.m. represents the average expression between 28 °C replicates. Products for oTSS represent the closest annotated gene

*w*MelPop-CLA				*w*AlbB			
**TSS type**	**Product**	**Locus tag**	**Mean c.p.m.**	**TSS type**	**Product**	**Locus tag**	**Mean c.p.m.**
iTSS	6S RNA	WD_RS06775	4.20E+05	iTSS	6S RNA	DEJ70_RS02130	1.45E+05
iTSS	Fructose-6-phosphate aldolase	WD_RS02455	2.93E+05	iTSS	Fructose-6-phosphate aldolase	DEJ70_RS05135	3.45E+04
pTSS	Hypothetical protein	WD_RS02845	8.41E+03	pTSS	16S rRNA	DEJ70_RS05685	1.46E+04
pTSS	Phage tail protein	WD_RS01240	8.12E+03	iTSS	Signal recognition particle sRNA small type	DEJ70_RS02165	1.02E+04
asTSS	DNA mismatch repair endonuclease MutL	WD_RS05920	5.39E+03	asTSS	DNA mismatch repair endonuclease MutL	DEJ70_RS05750	5.96E+03
iTSS	Signal recognition particle sRNA small type	WD_RS06780	3.58E+03	pTSS	Hypothetical protein	DEJ70_RS01070	5.67E+03
oTSS	Succinate dehydrogenase assembly factor 2	WD_RS04920	2.45E+03	iTSS	23S rRNA	DEJ70_RS05220	5.58E+03
pTSS	23S rRNA	WD_RS00885	2.25E+03	pTSS	Rne/Rng family ribonuclease	DEJ70_RS03135	5.57E+03
pTSS	16S rRNA	WD_RS05540	2.08E+03	pTSS	tRNA-Leu	DEJ70_RS01720	5.32E+03
pTSS	Thioredoxin family protein	WD_RS05680	2.04E+03	oTSS	Hypothetical protein	DEJ70_RS03120	5.00E+03
asTSS	Major capsid protein	WD_RS01225	1.95E+03	pTSS	Preprotein translocase subunit YajC	DEJ70_RS04650	4.18E+03
iTSS	Adenylosuccinate lyase	WD_RS03555	1.79E+03	pTSS	Hypothetical protein	DEJ70_RS01670	3.37E+03
pTSS	Bax inhibitor-1/YccA family protein	WD_RS04290	1.76E+03	pTSS	tRNA-Arg	DEJ70_RS04270	3.34E+03
pTSS	Hypothetical protein	WD_RS04725	1.64E+03	pTSS	Hypothetical protein	DEJ70_RS01345	3.20E+03
pTSS	Hypothetical protein	WD_RS00565	1.57E+03	pTSS	Hypothetical protein	DEJ70_RS01180	2.86E+03
pTSS	Ankyrin repeat domain-containing protein	WD_RS01925	1.56E+03	pTSS	Hypothetical protein	DEJ70_RS04380	2.78E+03
oTSS	Aspartate-semialdehyde dehydrogenase	WD_RS04305	1.23E+03	oTSS	ssDNA-specific exonuclease RecJ	DEJ70_RS01320	2.77E+03
pTSS	Hypothetical protein	WD_RS02135	1.17E+03	iTSS	tRNA-Ser	DEJ70_RS06770	2.65E+03
pTSS	TrbC/VirB2 family protein	WD_RS02935	1.10E+03	pTSS	DNA recombination protein RmuC	DEJ70_RS06410	2.48E+03
pTSS	Hypothetical protein	WD_RS00560	1.02E+03	pTSS	P44/Msp2 family outer membrane protein	DEJ70_RS02180	2.14E+03

### DE of pTSS

Sequencing depth for reads mapped to *Wolbachia* was sufficient for robust DE analysis in both strains, with the exception of the 34 °C condition at 48 h for *w*AlbB, which was excluded from the comparisons (Fig. S1). Analysis of the total number of reads mapped to various gene types, namely, protein-coding genes, rRNA, tRNA and other ncRNA, showed a relatively high proportion of reads mapped to the ncRNA category, which was comprised of only three genes (*ssrS*, *ffs* and *rnpB*) (Fig. S7). Despite this skewed distribution, the median number of reads for each gene across different categories was found to be sufficient for robust DE analysis (Fig. S8). In MDS plots, tight clustering of biological replicates by condition and timepoint was also apparent for the vast majority of samples (Fig. S9). We analysed total TSS from each strain for DE, and of the 966 and 4,318 TSS detected, 311 (32.2%) and 688 (15.9%) TSS were found to be differentially expressed (2-fold change, FDR 5%) under at least 1 temperature stress condition for *w*MelPop-CLA and *w*AlbB, respectively (Table 3, Fig. 2a; complete datasets are provided in Tables S2, S3). The proportion of TSS types that exhibited DE reflected the available TSS repertoire. Hence, for both *w*MelPop-CLA and *w*AlbB, gTSS represented the smallest group of DE TSS, whereas the largest group was pTSS or iTSS for *w*MelPop-CLA and *w*AlbB, respectively. Under antibiotic exposure, a total of 760 DE TSS (17.6%) were detected in *w*AlbB (Tables 3; S4).

**Table 3.** Summary of *w*MelPop-CLA and *w*AlbB differentially expressed TSS for each type between all stressors. Values represent the number of down- or upregulated (2-fold change, FDR 5%) TSS

	*Wolbachia* strain						
	***w*** **MelPop-CLA**		***w*** **AlbB**				
	No. of regulated TSS per stress condition* (down, up)						
**TSS category**	**21** **°C**	**34** **°C**	**21** **°C**	**34** **°C**	**Doxy.**	**Moxi**.	**Rif.**
**pTSS**	51, 16	135, 27	83, 16	122, 12	26, 40	2, 0	221, 8
**gTSS**	0, 0	6, 1	14, 0	22, 2	8, 2	2, 0	22, 0
**iTSS**	18, 6	65, 5	125, 9	166, 20	37, 26	19, 0	239, 15
**asTSS**	7, 3	35, 3	73, 7	99, 16	24, 15	7, 0	89, 27
**oTSS**	4, 5	14, 6	18, 13	22, 8	9, 3	1, 0	30, 8
**Total**	80, 30	255, 42	313, 45	431, 58	104, 86	31, 0	601, 58

*Doxy., doxycycline; Moxi., moxifloxacin; Rif., rifampicin.

Of the 175 *w*MelPop-CLA DE pTSS under temperature stress, 16 and 27 were upregulated at 21 and 34 °C, respectively, whereas of the 189 *w*AlbB DE pTSS, 16 and 12 were upregulated at 21 and 34 °C, respectively (Table 3). For both *w*MelPop-CLA and *w*AlbB, the majority of DE pTSS were downregulated in response to temperature stress (Table 3, Fig. 2a). Of the 760 *w*AlbB DE pTSS identified in response to antibiotic stress, 40, 0 and 8 were upregulated, while 26, 2 and 221 were downregulated under doxycycline, moxifloxacin and rifampicin stress, respectively (Table 3). Thus, in common with temperature stress, rifampicin exposure mostly induced downregulation of pTSS, whereas doxycycline exposure led to a greater proportion of upregulated pTSS (Fig. 2a). Surprisingly, the TSS expression patterns under moxifloxacin exposure were quite similar to control conditions (Fig. 2a), as reflected in the MDS plots (Fig. S9C).

**Fig. 3.**
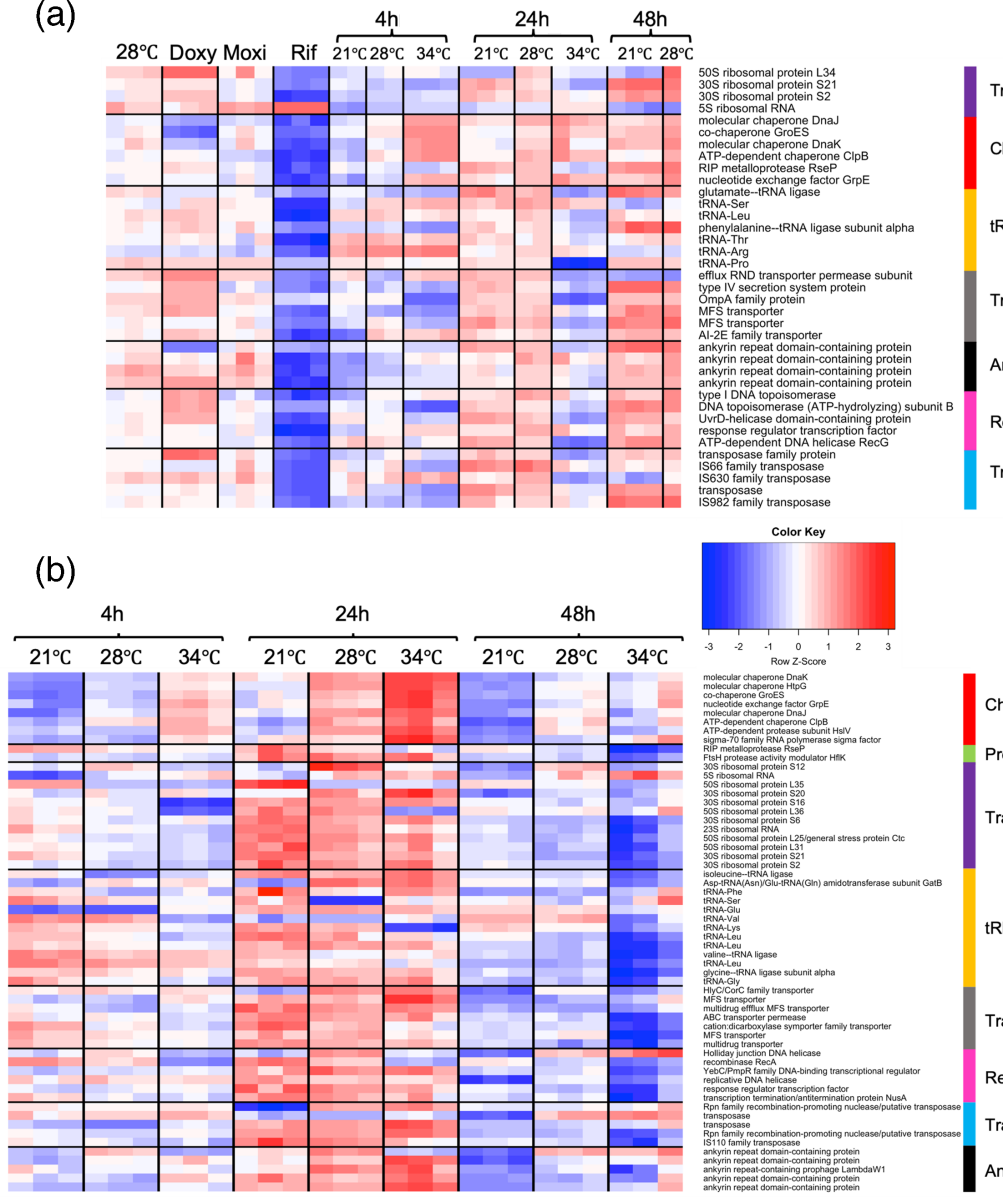
Summary heatmap of DE pTSS for (a) *w*AlbB and (b) *w*MelPop-CLA. Associated functional groups are displayed on the right. Expression levels represent *Z*-scores (sample c.p.m. minus mean c.p.m. of associated row, divided by sd of c.p.m. in that row).

To determine whether certain functional categories were overrepresented in the DE datasets, we applied KEGG pathway mapping. In both *w*MelPop-CLA and *w*AlbB, DE pTSS were associated with chaperones and heat-shock proteins (HSPs), translation, repair and recombination, nucleotide metabolism, lipid metabolism, mobile genetic elements, ankyrins, secretion and transport and carbon metabolism (Figs2b  3). The largest group of upregulated pTSS during temperature stress was chaperones and HSPs for both *w*MelPop-CLA and *w*AlbB (Figs.2b  3). Remarkably, pTSS associated with a phage portal protein pseudogene (DEJ70_RS00855) or transposase (WD_RS03395) was upregulated at 34 °C for *w*AlbB and *w*MelPop-CLA, respectively (Fig. 2b). The pTSS proximal to other mobile elements in *w*AlbB were affected by doxycycline treatment, as evidenced by upregulation of a transposase (DEJ70_RS06895), alongside downregulation of an apparent pseudogenized transposase (DEJ70_RS04660; Fig. 2b). Among antibiotic stressors, doxycycline induced the largest category of upregulated pTSS involved in secretion and transport (Fig. 3a).

**Fig. 4.**
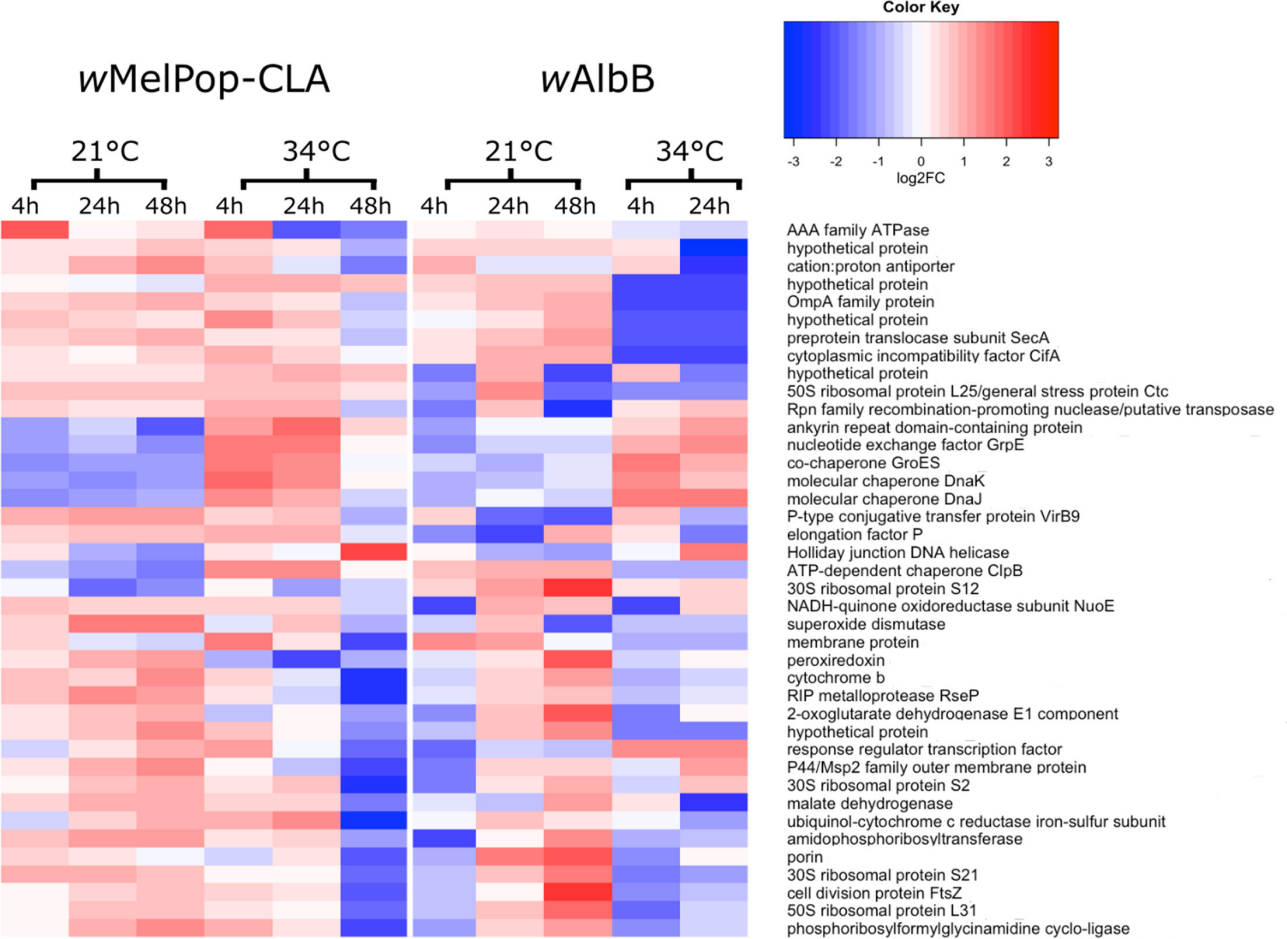
Heatmap of differentially expressed orthologous pTSS exposed to temperature stress. Orthologous pTSS were located upstream of genes encoding heat shock proteins, ankyrins, secretion systems and hypothetical proteins. The colour key is shared between strains, but the normalized value is specific to the individual strain. Expression levels are displayed in *Z*-scores (sample c.p.m. minus mean c.p.m. of associated row, divided by sd of c.p.m. of that row within each strain).

We identified orthologous genes shared by *w*MelPop-CLA and *w*AlbB pTSS (Fig. 2a) and analysed DE between pTSS of each strain (Table S5 and Fig. S10). Forty pTSS were classed as DE under at least one temperature stress condition (Fig. 4); these were located upstream of genes encoding HSPs (*dnaJ*, *groES*, *dnaK*, *dnaJ* and *htpG*), T4SS secretion components (*virB9*), translation machinery (ribosomal proteins), cell division apparatus (*ftsZ*), outer membrane proteins (OMPs; *ompA*) and hypotheticals. Both strains also exhibited regulation of heat shock-related protease genes such as *hslV* and *clpB*, with HSPs displaying upregulation at 34 °C and downregulation at 21 °C as expected. In contrast with *w*MelPop-CLA, *w*AlbB did not exhibit DE of the pTSS for *rpoH* (encoding σ^32^). Overall, the response of orthologous genes in each strain was more similar (Fig. S10) in the cold-shock comparison (21 °C vs. 28 °C) than in the heat-shock condition (28° vs. 34 °C).

### Putative sRNAs associated with antisense TSS

**Fig. 5.**
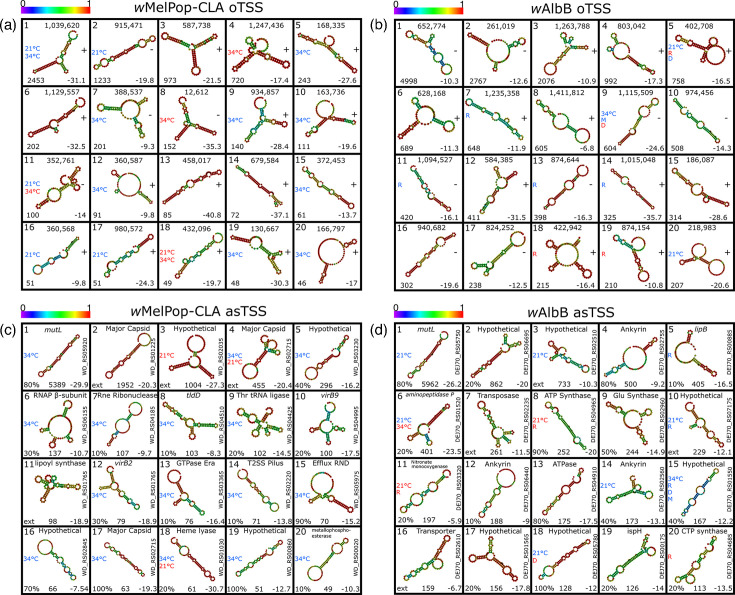
Summary of top 20 ncRNA and associated characteristics. Top 20 expressed oTSS for (a) *w*MelPop-CLA and (b) *w*AlbB. Top 20 expressed asTSS for (c) *w*MelPop-CLA and (d) *w*AlbB. Centre, predicted RNA secondary structure; top left, rank by expression; top centre, genome coordinate for oTSS or target gene for asTSS; right, strand for oTSS or locus for asTSS; bottom right, MFE value; bottom centre, average c.p.m. for asTSS; bottom left, average c.p.m. for oTSS or location along the gene as a percentage for asTSS; left, stressors which induced DE. Stressors are labelled as follows: R, rifampicin; M, moxifloxacin; D, doxycycline; 21 and 34 °C. Red- or blue-coloured text indicates up- or downregulation, respectively. Colour key spectrum above each panel represents base-pair probabilities.

To determine if asTSS may be associated with sRNA expression, the top 20 asTSS in *w*MelPop-CLA were sorted according to their average expression (Fig. 5c), which ranged from 49 to 5,389 c.p.m. The MFE values, which are inversely related to the stability of RNA secondary structures, were calculated for sRNAs predicted downstream of asTSS. These ranged from −7.5 to −30.7 at 28 °C. In contrast with iTSS and oTSS (Fig S11, Supplementary Material 1 and Table S6), the mean MFE of putative 100-bp sRNAs associated with asTSS (−15.5) was not significantly lower than the mean for randomized 100bp sequences from the *w*Mel genome (−14.7). Nevertheless, 17 of the top 20 asTSS from *w*MelPop-CLA displayed evidence of DE. For instance, *WsnRNA-59*, associated with a phage major capsid gene (Fig. 5c, rank 4), was upregulated over 5-fold at 21 °C and downregulated at 34 °C, while other asTSS, which may target a major capsid protein gene (WD_RS02715; Fig. 5c, rank 17) or *rpoB* (Fig. 5c, rank 6), were downregulated more than 2-fold under 34 °C exposure. Of the top 20 asTSS present in *w*AlbB, the average expression ranged from 113 to 5,962 c.p.m., whilst MFE values encompassed −5.9 to −26.2 (Fig. 5d), which was not significantly different from random for the *w*AlbB genome (−14.2). Unlike *w*MelPop-CLA, asTSS related to prophage genes were not prominently expressed in *w*AlbB, although one was associated with a major capsid pseudogene (Fig. S6, Supplementary Material 1). Evidence for DE was observed for 11 of the top 20 *w*AlbB asTSS under at least one stressor, especially for rifampicin exposure, which resulted in upregulation of six asTSS. An asTSS in opposite orientation to the mismatch repair protein gene *mutL* was the most highly expressed asTSS for both *w*MelPop-CLA (WD_RS05920) and *w*AlbB (Fig. S6, Supplementary Material 1), with an average expression of 5,389 and 5,962 c.p.m., respectively, at 28 °C. Both asTSS for *mutL* displayed DE, exhibiting downregulation at either 34 °C (*w*MelPop-CLA) or 21 °C (*w*AlbB). Unusually for *Pseudomonadota*, many *Wolbachia* genomes harbour two *mutL* paralogues [16], one of which is located in the eukaryotic association module of phage WO in strains containing prophages [51], such as *w*Mel and its derivatives. However, the asTSS was proximate to the *mutL* paralogue positioned outside prophage regions. In both *w*MelPop-CLA and *w*AlbB, RNA secondary structures with low MFE were predicted within the antisense sequence of the *mutL* gene, and the asTSS associated with *w*AlbB *mutL* was expressed at a 43-fold higher level than the pTSS in the sense orientation. Remarkably, the pTSS for the other *mutL* paralogue in *w*AlbB was poorly expressed, whereas in *w*MelPop-CLA, neither *mutL* paralogue had a detectable pTSS.

### Highly expressed intergenic and oTSS

An iTSS positioned within the *fsa* gene was consistently in the top two most highly expressed TSS for both *w*MelPop-CLA and *w*AlbB under all tested conditions but showed no evidence of DE (Fig. S6, Supplementary Material 1). The location of the *fsa* iTSS was identical for both *w*MelPop-CLA and *w*AlbB, suggesting high conservation and a common function. The *fsa* gene encodes fructose-6-phosphate aldolase, which catalyses the reversible formation of fructose 6-phosphate (F6P) from glyceraldehyde 3-phosphate and sedoheptulose 7-phosphate [52]. However, for F6P to be utilized for glycolysis, phosphorylation to fructose 1,6-bisphosphate by phosphofructokinase, which is absent in *Wolbachia*, is required. Moreover, the highly expressed iTSS of *fsa* was located downstream from the active site of the enzyme, such that if the transcript was translated into protein, it would be incapable of contributing to F6P synthesis. The RNA sequence 100 nt downstream of the *fsa* iTSS exhibited a secondary structure with low MFE in both *w*MelPop-CLA and *w*AlbB (Fig. S6, Supplementary Material 1), suggesting an alternative role as an ncRNA.

Of the 81 oTSS detected for *w*MelPop-CLA, the most highly expressed included 3 associated with *Wolbachia* ncRNAs predicted in an earlier study on *w*Mel [53]. The oTSS at position 1,039,620 (Fig. 5a, rank 1) was identified as *ncrwMel02* and was the most strongly expressed oTSS in this strain; moreover, the transcript was downregulated during exposure to temperatures of 21 °C or 34 °C. The small ncRNA designated *ncrwMel01* was detected at genomic position 587,738 (Fig. 5a, rank 3), while the third oTSS associated with a previously identified ncRNA from *w*Mel was located at position 67,860 (- strand), but exhibited very low expression. Although neither of these transcripts displayed evidence of DE, an oTSS located at genomic position 915,471 (Fig. 5a, rank 2) – incidentally not identified in the study of Woolfit *et al.* [53] – showed downregulation at 21 °C. In contrast, among the 310 oTSS discovered in *w*AlbB, very few highly expressed ncRNA candidates were regulated under thermal stress (3/20 compared with 16/20 in *w*MelPop-CLA; Fig. 5a and b).

### Predicted operon structures

We applied operon prediction to the genomes of both *Wolbachia* strains using TSS data. Of the 1,294 and 1,406 genes in *w*MelPop-CLA and *w*AlbB, respectively, 233 (18%) and 212 (15%) were predicted to be arranged into operons of 2 or more genes (Tables S7 and S8, Supplementary Material 1). The majority of operon-associated genes were predicted to be situated in gene pairs, representing 70% of operons in both *w*MelPop-CLA and *w*AlbB. The largest predicted operons were associated with the 30/50S ribosomal proteins, which contained 28 and 23 genes for *w*MelPop-CLA and *w*AlbB, respectively. Operons with a robustly located pTSS accounted for 17% (*n*=40) of the repertoire in *w*MelPop-CLA compared with 27% (*n*=58) in *w*AlbB. The least frequent TSS type associated with predicted operons was alternate gTSS, which accounted for just two and ten operons in *w*MelPop-CLA and *w*AlbB, respectively.

**Fig. 6.**
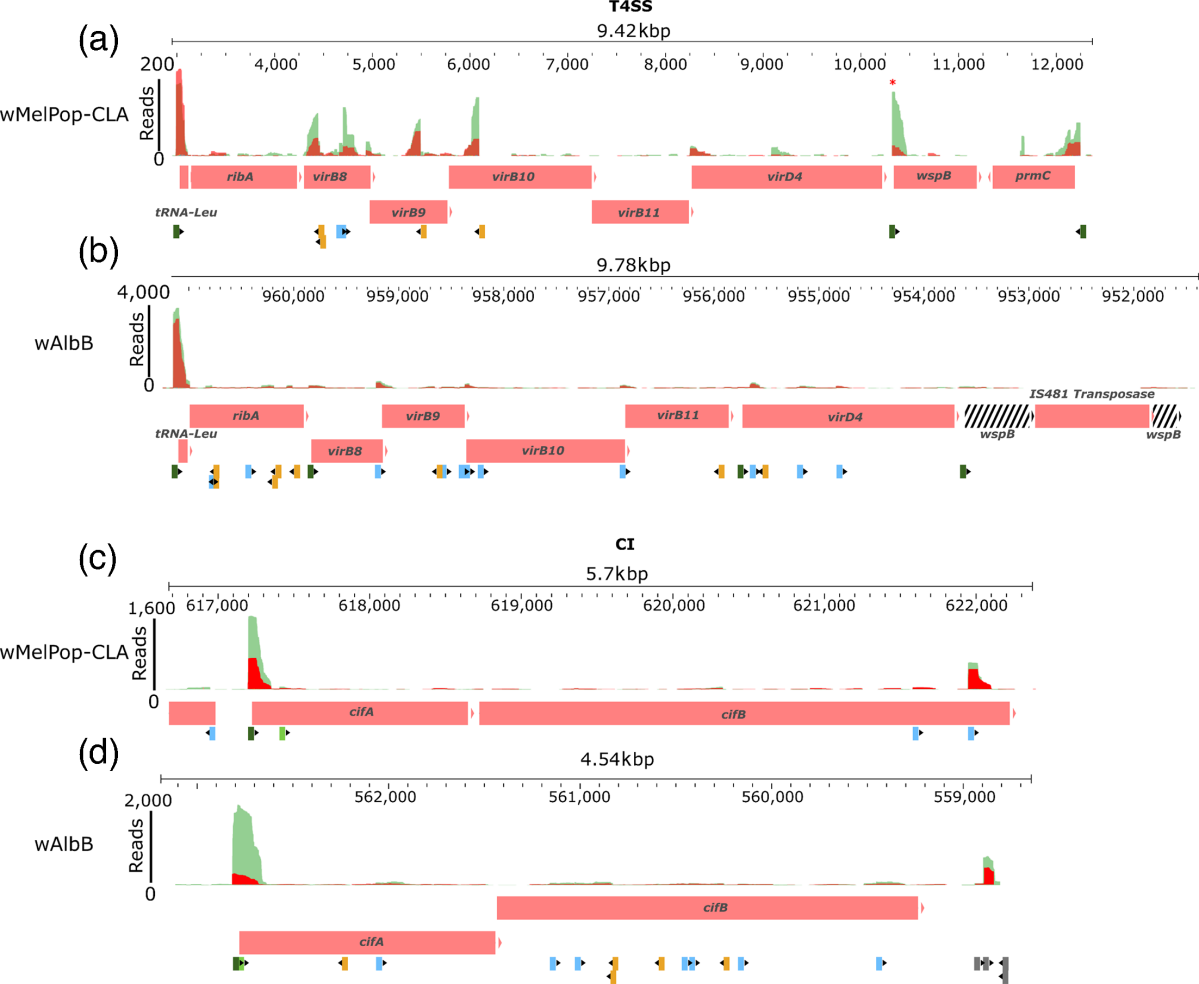
Overlay of TSS in relation to associated operons. The T4SS-1 operon for (a) *w*MelPop-CLA and (b) *w*AlbB. The CI operon for (c) *w*MelPop-CLA and (d) *w*AlbB. Genes are coloured as red boxes; pseudogenes are black and white striped. Green and red peaks represent mapped reads under 28 and 34°C temperatures, respectively. The TSS are indicated below the associated operon and coloured per TSS type: dark green, pTSS; light green, gTSS; light blue, iTSS; orange, asTSS; grey, oTSS. Red asterisk highlights the downregulation of *w*MelPop-CLA *wspB* during heat stress (34 °C).

The T4SS operons are of particular interest in *Wolbachia* biology due to the potential importance of secreted effectors in manipulating host cells, as well as the DE exhibited by some of its components for *w*AlbB, but not *w*MelPop-CLA, under temperature or antibiotic stress (Fig. 2b [20]). In both strains, the first operon (T4SS-1) is proximal to a surface protein gene [54], *wspB*, pseudogenization of which is associated with thermotolerance in natural genomic variants of *w*Mel and *w*AlbB strains [55,57]. We inspected the local region of the T4SS-1 operon, which contains eight genes from *tRNA^Leu^* – *wspB* in both *w*MelPop-CLA and *w*AlbB (Fig. 6a and b), and located a pTSS ~37 nucleotides upstream of the initiation codon of *tRNA^Leu^*. However, the TSS landscape across the operon displayed striking differences between the strains. In T4SS-1 of *w*MelPop-CLA (Fig. 6a), the operon contained a total of eight TSS, comprising two pTSS, two iTSS and four asTSS. The second pTSS for *w*MelPop-CLA was located in the intergenic region between *virD4* and *wspB*, ~20 nucleotides upstream of the *wspB* initiation codon. In contrast, the *w*AlbB-CLA T4SS-1 operon (Fig. 6b) contained a total of 23 TSS, comprising 3 pTSS, 11 iTSS, 8 asTSS and 1 pseudogene-associated pTSS (pTSSps). The three pTSS associated with the *w*AlbB T4SS-1 operon were upstream to *tRNA^Leu^*, *virB8* or *virD4*, whereas the pTSSps was 20 nucleotides upstream of the initiation codon for the *wspB* pseudogene.

We also examined the local region of the *cifA/cifB* CI operon, as limited data are currently available for its regulation in any strain. This revealed a single pTSS that was 25 and 32 nucleotides upstream of the *cifA* initiation codon in *w*MelPop-CLA and *w*AlbB, respectively, indicating a shared bicistronic arrangement (Fig. 6c, d). The *w*MelPop-CLA CI operon contained 4 TSS, comprising 1 pTSS, 1 gTSS and 2 iTSS, whereas the *w*AlbB CI operon contained 14 TSS (5 asTSS in addition to 1 pTSS, 1 gTSS and 7 iTSS). The *cifA* pTSS in *w*AlbB exhibited downregulation at 34 °C (Fig. 6d), while one of the asTSS associated with *cifB* displayed upregulation under rifampicin exposure, albeit from a very low baseline.

## Discussion

Prior to our study on *Wolbachia*, few studies on the primary transcriptome of obligate intracellular or other uncultivable bacteria had been performed. We previously applied a TEX-based protocol to *w*MelPop-CLA under doxycycline stress but did not analyse TSS distribution in that experiment [20]. Previous studies on *Chlamydia* spp. and *Candidatus* Phytoplasma asteris using the TEX method have identified several hundred TSS, equating to ~0.3–0.4 per kb [58,60]. This was surpassed by a TEX-based analysis of *Rickettsia conorii* TSS expression in tick and mammalian cell lines [61], which uncovered several thousand TSS (~3 per kb), almost identical to what we attained here for *w*AlbB. However, our study on *Wolbachia* was unique in applying replicated experiments across several stressors in two different strains. Indeed, a replicated Cappable-Seq study involving DE analysis of TSS across multiple stress conditions was achieved only recently in *Escherichia coli*, which identified 19,975 TSS (3.6 per kb) but was limited to a single strain [34].

The iTSS category was consistently the most poorly expressed type within both strains, suggesting that deeper sequencing depth preferentially uncovers more iTSS over other TSS classes. This may place limits on the extent to which deeper sequencing can uncover biologically relevant TSS. However, in *E. coli* – as here with *Wolbachia* – iTSS were consistently detected across biological replicates, and many exhibited DE between different conditions [34]. Thus, the total count of TSS for *w*MelPop-CLA would have been expected to be larger if the number of stress-inducing conditions was equivalent to those applied to *w*AlbB. While canonically, only a single origin of initiation upstream to the coding sequence of a gene is expected (i.e. a pTSS), the pervasiveness of iTSS we observed here is not limited to *E. coli*; other bacteria exhibiting high proportions of iTSS include *Mycobacterium marinum* (38%) [35], *Salmonella enterica* (11%) [36] and *Bacteroides thetaiotaomicron* (45%) [37].

For both *w*MelPop-CLA and *w*AlbB, the 6S RNA (encoded by *ssrS*) was consistently amongst the most highly expressed TSS. This ncRNA is known to form a hairpin secondary structure in which a pre-melted bubble mimics a DNA promoter capable of binding to the RNAP holoenzyme (Eσ). In *E. coli*, the bound 6S-Eσ complex results in downregulation of σ^70^ regulated genes by competitive binding to RNAP [62]. The expression of 6S RNA has previously been detected in both filarial and arthropod *Wolbachia* strains. Darby *et al*. [63] observed upregulation of 6S RNA from strain *w*Oo in the gonads of the filarial nematode *Onchocerca ochengi* relative to somatic tissue, whereas Darby *et al.* [20] reported downregulation of 6S RNA in the *w*MelPop-CLA strain exposed to doxycycline. The 6S rRNA of *w*AlbB was also shown to be upregulated in response to co-infection of mosquito cells by dengue virus *in vitro* [64], as well as in *R. conorii* during an infection time course in mammalian cells but not tick cells [61]. During the lifecycle of the filarial worm *B. malayi*, expression levels of 6S RNA in *Wolbachia* strain *w*Bm correlated with symbiont replication rates [65]. We did not observe regulation of *ssrS* in the current study, probably because 6S RNA acts as a template for product RNAs that are capped *in vivo* [66], and these processed transcripts would not be captured during the initial stage of the Cappable-Seq protocol. Few functional studies of 6S RNA have been performed in intracellular bacteria, but deletion of *ssrS* in *Legionella pneumophila* resulted in a tenfold reduction in intracellular growth along with downregulation of genes involved in AA metabolism and stress adaptation such as *groES* [67]. The 6S RNA is also essential for an effective oxidative stress response in *E. coli* [68]. The very high abundance of 6S RNA observed in the current study suggests that it not only controls intracellular replication but also prioritizes expression of stress-related genes, such as *groES*, with less reliance on sigma factors. Indeed, we only detected the presence of a sequence motif that resembled the −35 (TTGACA) and −10 (TATAAT) boxes of *E. coli* σ^70^, despite expression of σ^32^ in both *Wolbachia* strains and signs of its regulation in *w*MelPop-CLA, although this failure could be due to divergence of promoter motifs in the symbiont compared to free-living bacteria. An example of an unusual motif of unknown function was a TAAAAT sequence associated with gTSS or asTSS in both strains, which is a typical feature of the Pribnow box of Gram-positive bacteria [69] but was located 20–50 bases upstream of *Wolbachia* TSS. *Wolbachia* genomes may also encode unidentified extracytoplasmic functional sigma factors, as has been suggested for *Rickettsia* spp. [61].

Numerous studies have demonstrated that *Wolbachia* strains display marked differences in susceptibility to heat stress. Following early trials with the virulent strain *w*MelPop and its cell culture-adapted derivative, *w*MelPop-CLA, most field releases of *Wolbachia* for population replacement of *Aedes aegypti* worldwide have utilized *w*Mel due to its low fitness costs [70], although *w*AlbB is also well-tolerated by *Ae. aegypti* and has been the strain of choice for the Malaysian dengue control programme [71]. Both *w*MelPop-CLA and *w*Mel are sensitive to environmentally relevant heat stress, such as cycling of larval development temperatures from 26 to 37 °C [72]. Under laboratory conditions, this led to reduced symbiont density and loss of the CI phenotype for *w*MelPop-CLA, whereas *w*AlbB was significantly more tolerant and retained the ability to induce complete CI. However, data from the field after releases in Cairns, Queensland, showed that *w*Mel frequencies in transinfected mosquitoes can recover following a heatwave that reached 43.6 °C [73].

After being exposed to 34 °C heat stress for 4 h, both *w*MelPop-CLA and *w*AlbB upregulated HSP genes from the GroESL and KJE chaperone systems as expected. However, DE of *rpoH* under thermal stress was only apparent in *w*MelPop-CLA. Thus, *w*AlbB appears to upregulate HSP genes without, or with minimal contribution from, σ^32^. The heat-shock response peaked at 24 h, and by 48 h, *w*MelPop-CLA exhibited mass downregulation of all pTSS, including HSP genes, which may be indicative of cell death. Upregulation of *Wolbachia* HSPs during changes in the host cell environment has been observed previously in response to DENV infection [64] and host development [65,74]. Following cold stress at 21 °C, downregulation of GroESL or KJE components was apparent in *w*AlbB and *w*MelPop-CLA, respectively, reflecting an additional similarity in the two strains’ responses to temperature stress. Notably, it has been demonstrated that *w*Mel has co-adapted with *D. melanogaster* along latitudinal temperature clines in Australia and other continents, with a recently derived tropical variant exhibiting lower maternal transmission fidelity at cool temperatures (down to 20 °C) than does a temperate variant [55]. Unlike *w*MelPop-CLA, in which the *wspB* gene is intact, tropical variants of *w*Mel are associated with a premature stop codon at this locus, and strain wMelM maintained increased resilience to heat stress when transferred into *Ae. aegypti* [56]. We found a *wspB* pTSS that is independently regulated of the T4SS-1 operon of *w*MelPop-CLA; this ATU was downregulated at 34 °C. Similar to *w*Mel, two variants of *w*AlbB exist in the wild and exhibit alterations in thermal sensitivity that are associated with a polymorphism in *wspB* among other genomic differences [57], with the *wspB*-disrupted variant being used in culture here. Our data suggest that the pseudogenization of the *wspB* in *w*AlbB is accompanied by loss of regulation under temperature stress, although a remnant pTSS is associated with this disrupted ORF.

Despite their reduced genomes, *Wolbachia* strains from arthropods harbour an unusually high number of insertion sequences (IS), representing 6% or 13% of genomic content for *w*Mel and *w*AlbB, respectively [16,75]. Intact or degraded prophages originating from phage WO are also common in the arthropod symbionts. In *w*MelPop-CLA, we observed upregulation of a transposase gene at 34 °C, which progressively increased expression throughout exposure to elevated temperature, whereas doxycycline treatment induced upregulation of IS genes in *w*AlbB. Hence, similarly to phage WO in *Wolbachia*-infected *Nasonia vitripennis* wasps [76], some IS elements may be responsive to temperature increases, while upregulation of transposases as a result of antibiotic treatment has also been reported in free-living bacteria [77]. However, while the *w*MelPop-CLA genome contains two main prophage regions, no evidence of entry into a lytic cycle during heat stress was apparent here, as only a single phage structural component (phage tail protein, WD_RS02845) showed high expression from a pTSS. Instead, several asTSS associated with phage major capsid proteins were among the top 20 highly expressed asTSS in *w*MelPop-CLA.

Transcripts antisense to major capsid protein genes in the WO-A and WO-B prophage regions were previously observed in RNA-Seq studies on the susceptibility of *w*MelPop-CLA to doxycycline [20] and in *w*Mel during progression through the *D. melanogaster* lifecycle [21]. The antisense transcript overlapping the major capsid protein E gene in the WO-B prophage region was designated *WsnRNA-59* in a study of *Wolbachia*-host interactions [20,78]. The presence of these antisense transcripts to phage capsid protein genes suggests that *w*MelPop-CLA may be suppressing activation of the lytic cycle of phage WO via antisense–sense complementary degradation. The use of asRNA to inhibit the lytic cycle of phage has been observed in P22, hosted by *Salmonella* Typhimurium, which is repressed by the *c*_2_ protein that blocks transcription of proteins required for lytic cycle development [79]. Lytic growth of the P22 phage is activated by its anti-repressor protein (Ant) that counteracts *c*_2_ repression. However, a phage-encoded asRNA, Sar, can prevent the lytic cycle by blocking the *ant* ribosome binding site, inhibiting translation of the anti-repressor [80]. *Salmonella* Typhimurium also expresses an asRNA, Isrg, levels of which were inversely correlated with expression of a phage tail component [81]. Accordingly, the presence of genes encoding the dsRNA-specific RNase III in both *w*MelPop-CLA and *w*AlbB is consistent with a potential gene-silencing mechanism. In addition to phage-related asRNA, several transposase genes were associated with asTSS in both *Wolbachia* strains. Recently, antisense transcription has been hypothesized to suppress expression of *Rickettsiales*-amplified genetic elements (RAGEs) at the protein level in *Orientia tsutsugamushi*, a pathogen in the same order as *Wolbachia* (*Rickettsiales*), since RAGE products with high ratios of antisense to sense transcription were not detectable by proteomics in contrast with those exhibiting low ratios [82].

Phage portal proteins are key initiators of capsid assembly [83]; therefore, the DE associated with portal pseudogenes observed here may reflect regulatory remnants of previously active phages in the evolutionary history of *Wolbachia* that have yet to be completely lost. Pervasive transcription from pseudogenes (not limited to mobile elements) has also been reported from bacteria with more extensively degraded genomes than those of arthropod *Wolbachia*, such as *Mycobacterium leprae* and the tsetse fly symbiont, *Sodalis glossinidius*. In *M. leprae*, some pseudogenes have retained functional promoters [84], while in *S. glossinidius*, DE involving pseudogenes has been observed [85]. Thus, expression from a pTSS for the *wspB* pseudogene in *w*AlbB indicates that it may have a regulatory role associated with the T4SS-1 operon.

While the genes responsible for CI were identified several years ago (*cifA* and *cifB* in *w*Mel [86]), with homologues referred to as *cidA* and *cidB* in other strains [87], their regulation remains poorly understood. A key question is whether their conserved orientation as a gene pair represents evidence of expression as a bicistronic operon [88,89], which has been challenged by the large disparity in expression levels between the individual genes (i.e. an eightfold higher expression of *cifA* than *cifB* in *Drosophila in vivo*) [90]. In the present study, a single pTSS was identified upstream of *cifA* for both *w*MelPop-CLA and *w*AlbB, with no pTSS detected within the intergenic region in *w*MelPop-CLA or upstream of *cifB* in *w*AlbB. This supports a model in which *cifA* and *cifB* can be co-transcribed as a bicistronic transcript as previously determined from reverse transcription PCR evidence, despite the substantial difference in abundance between the individual transcripts. Lindsey *et al*. [90] identified a Rho-independent transcription terminator within the *w*Mel *cifA*–*cifB* intergenic region that could provide a mechanism to regulate the two genes if the activity of the terminator is partial. Other potential mechanisms that could reconcile the data supporting co-transcription and differential regulation between *cifA* and *cifB* are the presence of a Puf family-like RNA-binding domain within the *cifA* gene [90] and/or the use of alternative TSS. Although expression of the *cifB* asTSS in *w*AlbB was weak under the conditions tested here and not conserved in *w*MelPop-CLA, it highlights the complexity of regulation of operons and the possibility of ‘fine tuning’ of *cifA* and *cifB* expression, which in whole organisms may be temporal, tissue-specific or calibrated by host sex.

Since the late 1990s, extensive efforts have been made to develop antibiotic regimens that can safely eliminate adult filarial worms in onchocerciasis and lymphatic filariasis by targeting their *Wolbachia* symbionts, which, in contrast with most arthropod *Wolbachia* strains, are essential for the viability and reproductive physiology of their hosts [12]. Initially, doxycycline (a tetracycline derivative) garnered the most interest, but regimens of several weeks are required for killing of adult worms, which led to evaluation of antibiotics from a variety of other classes (e.g. rifamycins, fluoroquinolones and macrolides), some of which clear *Wolbachia* more rapidly than doxycycline in preclinical assays [40]. As no stable culture system for filarial endosymbionts has been established, arthropod *Wolbachia* strains (especially *w*AlbB) in mosquito cell lines have been utilized as a substitute [33,91].

Very few studies have attempted to characterize the global response of *Wolbachia* to antibiotic stress, especially in arthropod strains. However, we previously performed RNA-Seq in a TEX experiment (combined with proteomics) on *w*MelPop-CLA following exposure to doxycycline *in vitro* for 3 days [20]. Many similarities were apparent between wMelPop-CLA in the TEX study and the current Cappable-Seq study of *w*AlbB after 24 h exposure to doxycycline. These included downregulation of HSP genes and upregulation of ribosomal protein genes, enzymes involved in nucleotide or lipid metabolism, the twin-arginine translocation system and transposase genes. There were also a few differences, such as upregulation of some OMP genes and an ankyrin (both downregulated in *w*MelPop-CLA), whereas genes of the T4SS were upregulated in *w*AlbB but unaffected by doxycycline in *w*MelPop-CLA. Importantly, this does not necessarily indicate dissimilar responses between strains, as both the primary transcript enrichment method (TEX *v*. Cappable-Seq) and the antibiotic exposure period (1 or 3 days) are likely to have had a considerable impact on the comparability of the datasets.

While there were distinctive TSS expression profiles and DE associated with antibiotic stress compared with temperature stress, there were also areas of overlap, such as downregulation of HSP genes following cold stress or exposure to doxycycline. Indeed, responses to antibiotics are known to stem from conserved bacterial stress response networks, with tetracyclines inducing a cold-shock response in *E. coli* that includes downregulation of *rpoH* (though some HSP genes are nevertheless upregulated), as well as synergistic effects on *E. coli* viability when combined with cold stress [92]. Doxycycline-induced ribosomal stalling produces incompletely synthesized polypeptides that are then recycled via protease activity [93]. Doxycycline exposure induced upregulation of a gene encoding an inner membrane-bound protease, *ftsH*, in *w*AlbB, which contributes to the degradation of proteins for AA recycling [94]. FtsH-mediated proteolysis is also involved in the post-translational regulation of the heat-shock regulator RpoH [95]. Thus, proteolytic degradation of RpoH by FtsH could contribute to downregulation of heat-shock genes whilst providing an AA pool for nutritional uptake.

Changes to the structure of the Gram-negative outer membrane and activity of transporters spanning the periplasmic space are common during exposure to antibiotics, which can be accompanied by alterations to phospholipid metabolism. A pTSS associated with the *plsC* gene (DEJ70_RS02625), which encodes 1-acyl-sn-glycerol-3-phosphate acyltransferase of the glycophospholipid metabolism pathway, was the most upregulated pTSS in response to doxycycline exposure. PlsC is an integral membrane protein responsible for the catalytic conversion of 1-acyl-sn-glycerol-3-phosphate into 1,2-diacyl-sn-glycerol 3-phosphate (phosphatidic acid) [96], which in turn is the major precursor for membrane phospholipids such as phosphatidylethanolamine, phosphatidylglycerol and cardiolipin. In parallel, upregulation of genes encoding a major facilitator superfamily (MFS) transporter, a resistance-nodulation-cell division (RND) permease subunit and the outer membrane efflux protein TolC was observed. Both MFS transporters and the efflux pumps of the RND superfamily are associated with multidrug resistance in *E. coli* and other Gram-negative bacteria [97,99]. TolC is a component of tripartite efflux pumps, including the RND superfamily, MFS transporters and the type I secretion system. In the latter, HlyD (also upregulated in response to doxycycline) is an adaptor that facilitates transport of large proteins across the periplasmic space [98]. In *E. coli*, mutations in *tolC* led to a five- to sixfold decrease in tetracycline resistance conferred by Tet(A) pumps, which was demonstrated to result from disruption to an RND efflux system [100]. Only a single gene associated with secretion or transport, *yidC*, was downregulated after doxycycline transport, which encodes a membrane protein insertase. Notably, in *Aeromonas hydrophila*, knockout of *yidC* substantially lowered susceptibility to several antibiotics of the tetracycline class [101].

Further evidence for membrane remodelling following doxycycline exposure was apparent from upregulation of pTSS associated with OMPs such as peptidoglycan-associated lipoprotein (PAL), a member of the OmpA-like family and the primary pathogen-associated molecular pattern in *Wolbachia* cells [102]. This OMP has been partially characterized in *E. coli* and other Gram-negative pathogens such as *Pseudomonas aeruginosa* (where the PAL homologue is OprL) and *Haemophilus influenzae* (homologue is P6). Close associations between PAL and OmpA itself (or its homologues) confer integrity to the outer membrane [103], and mutants lacking P6 in *H. influenzae* have greatly increased susceptibility to antibiotics [104]. Few RNA-Seq studies have been performed on Gram-negative bacteria exposed to doxycycline, but in *Yersinia pseudotuberculosis*, two OMP genes (including a lipoprotein gene, although not a PAL homologue) were the only genes found to be upregulated following treatment and were associated with doxycycline tolerance [105].

Rifampicin (a rifamycin targeting RNAP) or moxifloxacin (a fluoroquinolone targeting DNA gyrase and topoisomerase IV) had a much more limited impact on DE of TSS in the 24 h timescale of our experiments compared with doxycycline. Over the past 25 years, the susceptibility of *Wolbachia* to various antibiotics (including rifampicin and moxifloxacin) has been established *in vitro* by adding compounds to mosquito cell lines infected with insect-derived *Wolbachia* strains, or by incubation of adult filarial worms in culture media containing drugs [33]. Cell line-based studies have differed between laboratories in whether *Wolbachia* are inoculated into host cells simultaneously with, or prior to, drug exposure, which leads to different growth kinetics [40]. In the Aa23 cell line, the doubling time of *w*AlbB has been estimated as 14 h [41], while in adult filarial worms, turnover of symbiont populations is very slow except in the female reproductive system [106]. Antibiotic susceptibility testing is performed over a period of at least several days, sometimes with replenishment of drugs midway through the assay [40]. In our study, we harvested cells 24 h after antibiotic exposure, since a longer treatment period would have increased the proportion of dead bacteria and reduced *Wolbachia* RNA yields. Similarly, host cells were sub-cultured several days prior to treatment to ensure that *Wolbachia* density had plateaued at a level permitting recovery of sufficient symbiont RNA for high transcriptome sequence coverage. In contrast, RNA-Seq experiments on free-living bacteria exposed to fluoroquinolones added the antibiotic to liquid medium during the mid–late exponential phase [107,108]. This may explain why by 24 h post-treatment, we did not observe any signs of the global SOS response in *Wolbachia* exposed to moxifloxacin, whereas SOS induction is typical in free-living bacteria following short-term incubation with fluoroquinolones.

Although the number of TSS exhibiting DE after rifampicin treatment was small, it was clear that this drug had an opposite effect to doxycycline on genes involved in secretion and transport. Rifampicin triggered downregulation of T4SS components and an RND transporter permease (both upregulated by doxycycline), whereas *yidC* was upregulated by rifampicin but downregulated by doxycycline. These results underscore specific aspects of regulation in *Wolbachia* following exposure to stress within a larger framework of stress-responsive genes. Studies in *E. coli* have shown that while rifampicin blocks the activity of free RNAP molecules, those already bound to DNA and participating in transcription are not inhibited, permitting residual RNA synthesis [109]. Thus, the lack of broadscale downregulation of *Wolbachia* transcription by 24 h observed here is compatible with persistent RNAP activity at TSS across the genome. Experiments designed to characterize the non-culturable state in *Mycobacterium tuberculosis* use potassium ion depletion to induce a dormant state followed by rifampicin treatment, which kills dividing bacilli but not dormant persisters. However, ongoing transcriptional activity in dormant *M. tuberculosis* is thought to result from greatly extended transcript stability rather than ongoing transcription *de novo* under drug pressure [110]. Notably, in long-term experiments in animal models of filariasis, rifampicin failed to completely clear *Wolbachia* symbionts [111], leading to recrudescence from persisting populations in the reproductive tract of female worms in the absence of mutations in the *Wolbachia* genome [112]. Hence, these filarial *Wolbachia* displayed rifampicin tolerance rather than resistance. One candidate molecule that might contribute to persistence is RNase P, a ribozyme that is essential for the processing of precursor tRNA transcripts, which was upregulated after rifampicin exposure in *w*AlbB. Accordingly, an increase in RNase P levels after rifampicin treatment has also been observed in *Bacillus subtilis* [113].

## Conclusions

This study detected statistically significant DE amongst all known TSS types for both *w*MelPop-CLA and *w*AlbB. This highlights the severe limitations of conventional RNA-Seq, in which distinct transcriptional units are merged and quantified in aggregate. Moreover, it suggests that the characterization of *Wolbachia* (and other obligate intracellular bacteria) as organisms with limited capacity for transcriptional regulation is premature [19], reflecting technology and not biology. At a minimum, the sophisticated reproductive manipulations induced by arthropod *Wolbachia* strains are likely to be stringently controlled between host tissues and sensitive to host sex [21,114]. However, the transcriptional landscape should also be revisited in the filarial symbionts with more reduced genomes to determine their TSS repertoire and potential for regulation across the lifecycle, and/or between the somatic and germline cells of the host [63,115]. This is particularly important for understanding transcriptional plasticity under drug pressure [20] and its relationship to the challenges of eliminating *Wolbachia* with short antibiotic regimens for the control of filarial diseases.

Our data show that the differences in thermal susceptibility observed between *w*AlbB and *w*MelPop-CLA [72] are not due to a failure by the latter to induce a heat-shock response; indeed, only *w*MelPop-CLA displayed regulation of *rpoH* following temperature stress. Instead, differences in host background, specifically hosts that share a longer phylogenetic history with their symbiont, may impact host-symbiont homeostasis during stress conditions. While the *wspB* polymorphisms previously identified [55,57] play a key role in thermal sensitivity, future studies should focus on how the differential regulation of ncRNAs and transposase activation impacts the resilience of *Wolbachia* strains exposed to heat and other stressors. Applying Cappable-Seq to *Wolbachia* in whole invertebrate host systems will provide further insights into this remarkably adaptable and successful symbiont.

## Supplementary material

10.1099/mgen.0.001542 Supplementary Material 1.

10.1099/mgen.0.001542 Uncited Table S1.

10.1099/mgen.0.001542 Uncited Table S2.

10.1099/mgen.0.001542 Uncited Table S3.

10.1099/mgen.0.001542 Uncited Table S4.

## Abbreviations

asTSS: antisense TSS
ATU: alternative transcriptional unit
CI: cytoplasmic incompatibility
DE: differential expression
DTB-GTP: 3′-desthiobiotin-guanosine triphosphate
F6P: fructose 6-phosphate
gTSS: gene-associated TSS
HSPs: heat-shock proteins
IS: insertion sequences
iTSS: intragenic TSS
KEGG: Kyoto Encyclopedia of Genes and Genomes
KO: KEGG Ortholog
MDS: multidimensional scaling
MFE: minimum free energy
MFS: major facilitator superfamily
ncRNA: non-coding RNA
NEB: New England Biolabs
OMPs: outer membrane proteins
oTSS: orphan TSS
PAL: peptidoglycan-associated lipoprotein
5′PPP: 5′-triphosphate
pTSS: primary TSS
pTSSps: pseudogene-associated pTSS
RACE: rapid amplification of cDNA ends
RAGEs: Rickettsiales-amplified genetic elements
RND: resistance-nodulation-cell division
sRNAs: small RNAs
TEX: terminator 5′-phosphate-dependent exonuclease
TSS: transcriptional start sites
