# Erratum: Cappable-seq reveals the transcriptional landscape of stress responses in the bacterial endosymbiont Wolbachia

**DOI:** 10.1099/mgen.0.001584

**Published:** 2025-12-02

**Authors:** Youseuf Suliman, Zhiru Li, Amit Sinha, Philip D. Dyer, Catherine S. Hartley, Laurence Ettwiller, Alistair C. Darby, Clotilde K. Carlow, Benjamin L. Makepeace

**Affiliations:** 1 Institute of Infection, Veterinary & Ecological Sciences, University of Liverpool, Liverpool, Merseyside, L3 5RF, UK; 2 New England Biolabs, Ipswich, MA, 01938, USA

In the published version of this article, an error was identified in one of the figures. Figure 3 had been partially cut off, which resulted in the labels on the right side not being visible. The correct figure is provided below and has been updated in the original article.

In addition, in the second paragraph on page 8, the citation at the end of the sentence should refer to Fig. S5 rather than Fig. S4. The corrected sentence is as follows:

‘Analysis of motifs within the various subtypes of TSS also identified these promoter motifs in pTSS, iTSS and oTSS in both wMel and wAlbB, while other motifs such as TAAAAT were found for gTSS and asTSS (Fig. S5).’

This has also been updated in the original article.

The Microbiology Society apologises for any inconvenience caused.

