# Supplementary material for: Cappable-Seq reveals the transcriptional landscape of stress responses in the bacterial endosymbiont Wolbachia
Source: Microb Genom. 2025 Oct 27;11(10):001542. doi: 10.1099/mgen.0.001542 (PMC12558409; doi:10.1099/mgen.0.001542)
Supplement: Supplementary Material 1. [file mgen-11-01542-s001.pdf]

Cappable-Seq reveals the transcriptional landscape of stress responses in the bacterial endosymbiont *Wolbachia*

Youseuf Suliman[1], Zhiru Li[2], Amit Sinha[2], Philip D. Dyer[1], Catherine S. Hartley[1], Laurence Ettwiller[2], Alistair C. Darby[1], Clotilde K. Carlow[2], Benjamin L. Makepeace[1*]

[1]Institute of Infection, Veterinary & Ecological Sciences, University of Liverpool, Liverpool, Merseyside, L3 5RF, UK

[2]New England Biolabs, Ipswich, MA, 01938, USA

* To whom correspondence should be addressed. Tel: +44 151 7941586 Email: blm1@liverpool.ac.uk

- Supplementary Figures S1 to S11
- Supplementary Tables S1 and S6 to S8

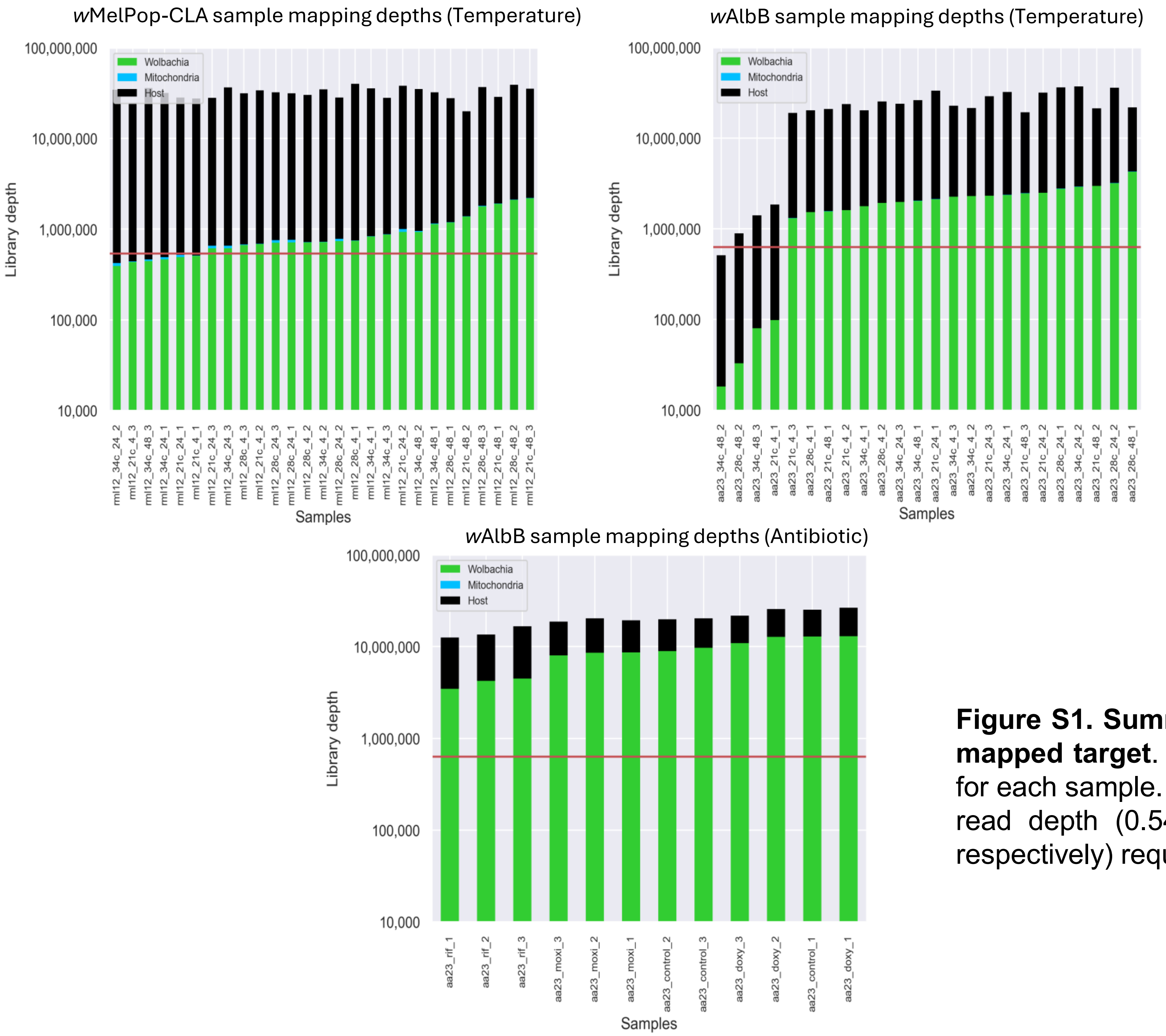

Figure S1. Summary of mapped reads across conditions and their mapped target. Bar chart representing reads depths of mapped reads for each sample. Red horizontal line represents the suggested minimum read depth (0.54M and 0.63M reads for *w*MelPop-CLA and *w*AlbB respectively) required for confident differential expression analysis.

Table S1 5’ RACE primer sequences.

Gene ID	Gene	Strain	Primer sequence
DEJ70_RS04995	*rpsM*	*w* AlbB	Inner: TTTCTGAGCTCACCCTCTATGACA
DEJ70_RS04995	*rpsM*	*w* AlbB	Outer: AGCATTAGTATGGGTTCTTTGTCCCC

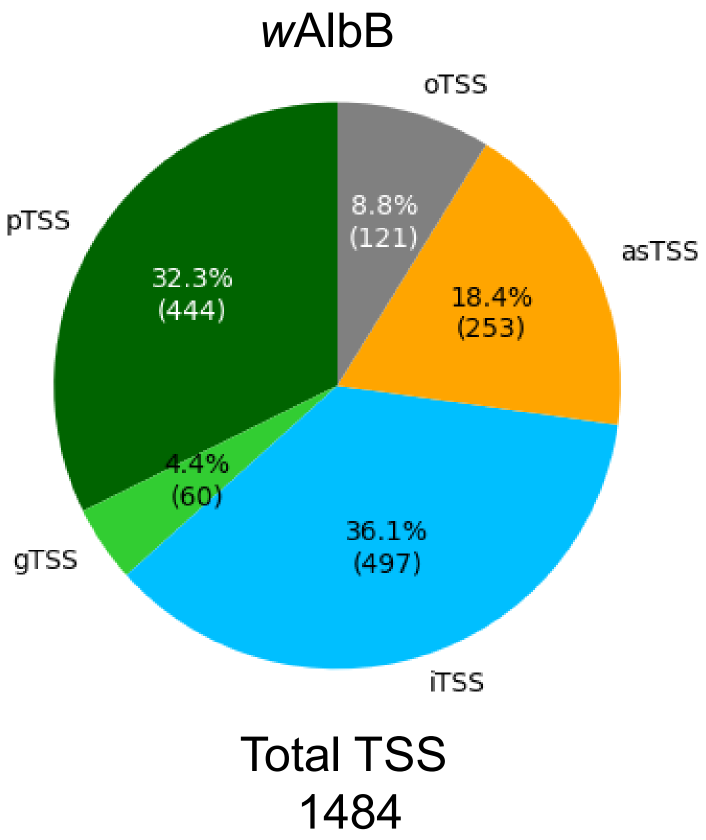

Figure S2. Summary of *w*AlbB TSS types across all conditions tested using the CPM threshold identical to *w*MelPop-CLA (25.48 CPM) representing the CPM of 10 reads in the smallest library that passed the recommended depth (0.54M and 0.63M reads for *w*MelPop-CLA and *w*AlbB respectively).

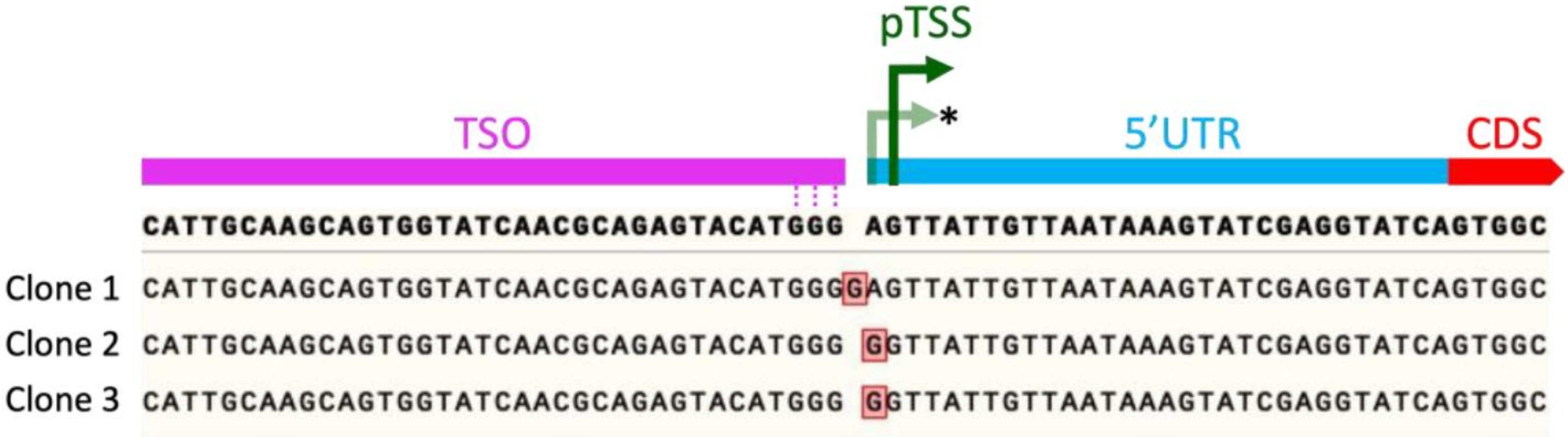

Figure S3. TSS validation with 5' RACE. The pTSS of *w*AlbB *rpsM* was validated using a template switching 5' RACE protocol. Segments shown are the template switching oligonucleotide sequence with 3' poly-G tail (pink) followed by the pTSS discovered by Cappable-Seq (dark green), 5' UTR (blue), and the initial 5 nucleotides of the uS13 coding sequence.

*Represents an adjacent minor pTSS one nucleotide upstream. Three 5' RACE sequences derived from separate clones are displayed; the highlighted guanosine (G) nucleotide represents a known non-template addition.

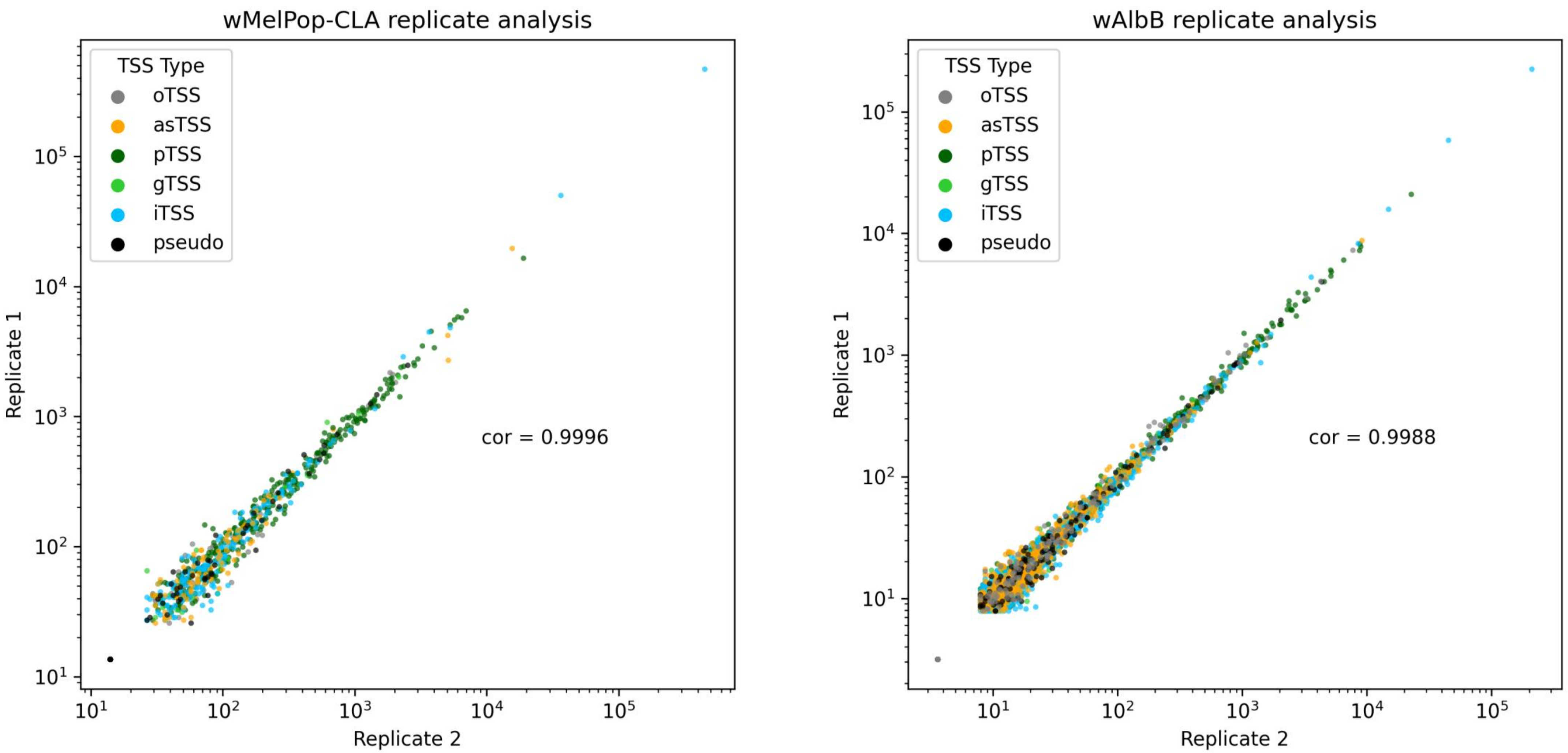

Figure S4. Cappable-seq expression between replicates. Scatter graphs of TSS expression between 28℃ replicates of A) *w*MelPop-CLA and B) *w*AlbB. Each dot represents the CPM of each TSS coloured by its designated TSS type. cor = correlation coefficient.

Figure S5. Motif analysis in the 100 bp upstream regions of various TSS sub-types.

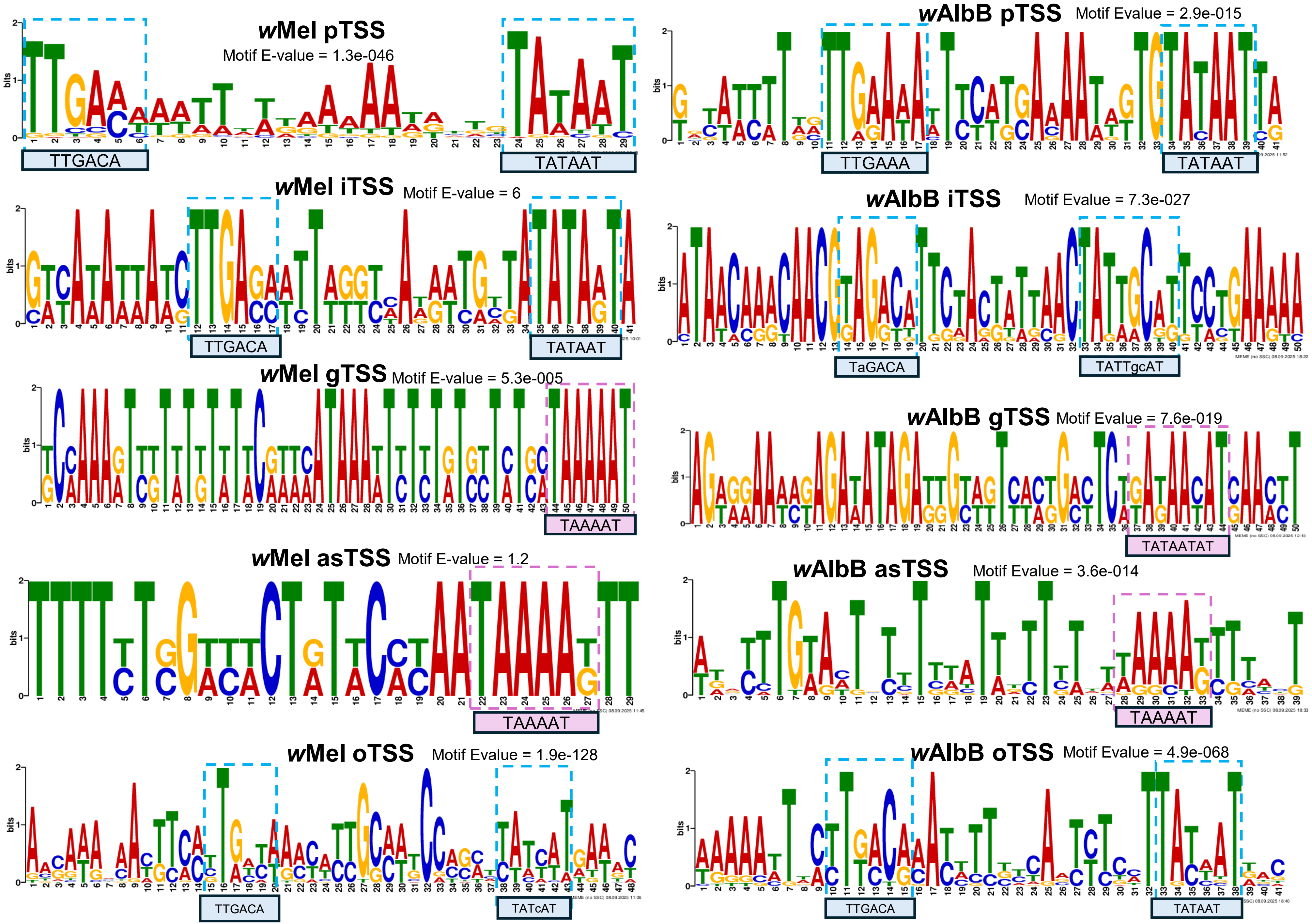

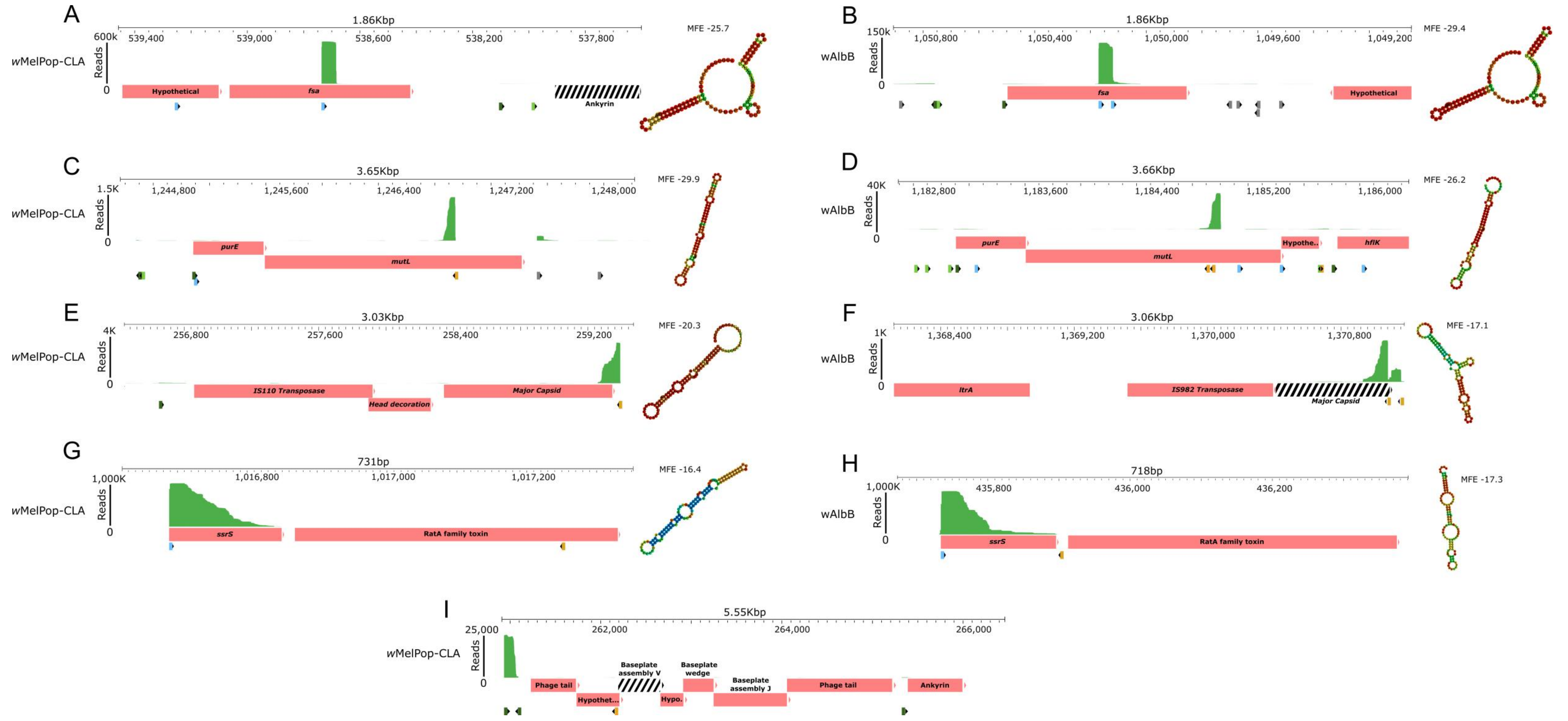

Figure S6. Overlay of TSS on select regions of interest. The iTSS of *fsa* for **A** *w*MelPop-CLA and **B** *w*AlbB. The asTSS of *mutL* for **C** *w*MelPop-CLA and **D** *w*AlbB. The asTSS of the major capsid for **E** *w*MelPop-CLA and **F** *w*AlbB. The iTSS of *ssrS* (6S) for **G** *w*MelPop-CLA and **H** *w*AlbB. Genes are coloured red boxes, pseudogenes are black and white striped. Green peaks represent mapped reads under 28° C. TSS placed below associated genes and coloured per TSS type as dark green, pTSS; light green, gTSS; light blue, iTSS; orange, asTSS; grey, oTSS. Predicted RNA secondary structures for the 100nt downstream sequence are placed to the right of each displayed region along with the associated MFE value.

Discussion of Fig. S6

The identity of some of the most highly-expressed alternative TSS was surprising, such the asTSS associated with a *mutL* paralogue, which was the dominant antisense transcript in both strains. When interpreted in the context of poor *mutL* pTSS expression, this suggests suppression of mismatch repair in *Wolbachia*. Mutations in *mutL* have been shown to increase conjugal recombination frequencies between *E. coli* and *Salmonella* Typhimurium by 1,000-fold[1], indicating that antisense regulation of *mutL* in *Wolbachia* might explain genome complexity in arthropod strains, which harbour elevated proportions of mobile elements compared to most other obligate intracellular symbionts[2,3]. The very highly expressed iTSS associated with the *fsa* gene was also unexpected and the lack of enzymatic conserved domain encoded by this transcript is strongly suggestive of an ncRNA-mediated regulatory role. Importantly, a limitation of Cappable-Seq is that the 3' end of transcripts are not captured due to short read lengths and a single-end library protocol, preventing identification of full-length molecules and restricting structural predictions to the first 100 nt. Nevertheless, detection of TSS associated with ncRNAs reported in previous studies, such as the oTSS linked with *ncrwMel02*[4], provided corroboration that our approach revealed genuine ncRNAs and not biological noise. Only two *Wolbachia* ncRNAs have had their lengths experimentally confirmed by Northern blot, *WsnRNA-46* and *WsnRNA-59,* for which the precursor transcript sizes were ~70 bases and the length of the mature molecules was ~30 bases[5]. However, ncRNAs such as *ncrwMel02* may be longer than this according to previous studies using 5' RACE and RT-PCR with *w*Mel[4]. In whole flies, *ncrwMel02* was upregulated in testes relative to ovaries[4], suggesting a role in host reproductive manipulation, while we detected downregulation of this transcript under temperature extremes. This may be pertinent to the well-known influence of temperature on the phenotype of CI in *Drosophila*[6].

[1]**Matic I, Rayssiguier C, Radman M**. Interspecies gene exchange in bacteria: The role of SOS and mismatch repair systems in evolution of species. *Cell*. 1995; doi: 10.1016/0092-8674(95)90501-4.

[2]**Cordaux R, Pichon S, Ling A, Pérez P, Delaunay C, Vavre F, *et al*.** Intense transpositional activity of insertion sequences in an ancient obligate endosymbiont. *Mol Biol Evol*. 2008; doi: 10.1093/molbev/msn134.

[3]**Kaur R, Shropshire JD, Cross KL, Leigh B, Mansueto AJ, Stewart V, et al**. Living in the endosymbiotic world of *Wolbachia*: A centennial review. *Cell Host Microbe*. 2021; doi: 10.1016/j.chom.2021.03.006.

[4]**Mayoral JG, Hussain M, Joubert DA, Iturbe-Ormaetxe I, O'Neill SL, Asgari S**. *Wolbachia* small noncoding RNAs and their role in cross-kingdom communications. *Proc Natl Acad Sci*. 2014; doi: 10.1073/pnas.1420131112.

[5]**Woolfit M, Algama M, Keith JM, McGraw EA, Popovici J.** Discovery of putative small non-coding RNAs from the obligate intracellular bacterium *Wolbachia pipientis*. *PLoS ONE*. 2015; doi: 10.1371/journal.pone.0118595.

[6]**Clancy DJ, Hoffmann AA**. Environmental effects on cytoplasmic incompatibility and bacterial load in *Wolbachia*-infected *Drosophila simulans*. *Entomol Exp Appl*. 1998; doi: 10.1046/j.1570-7458.1998.00261.x.

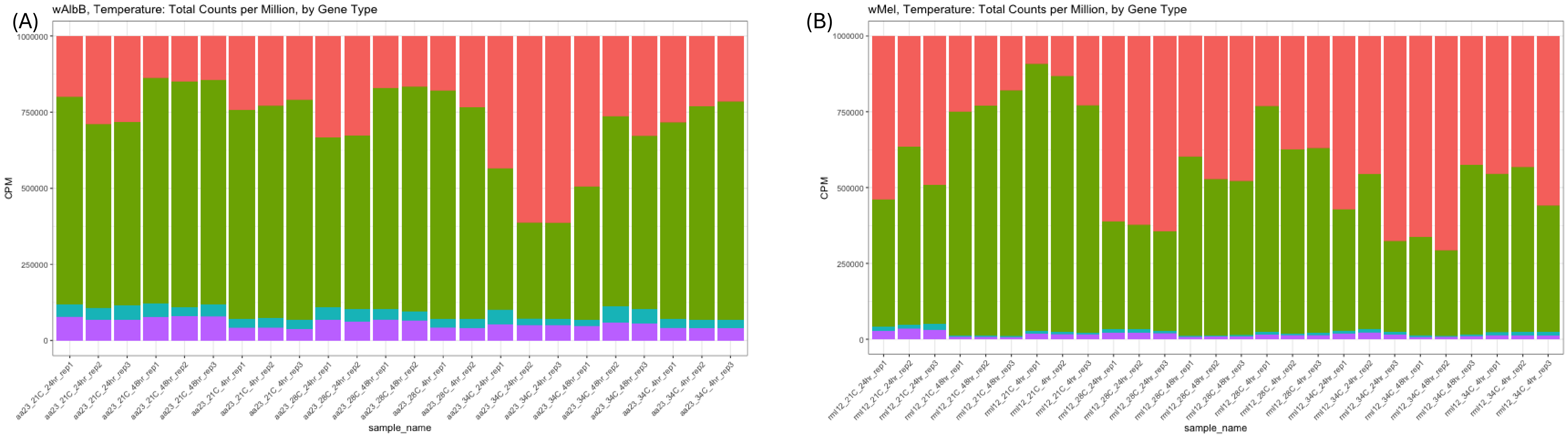

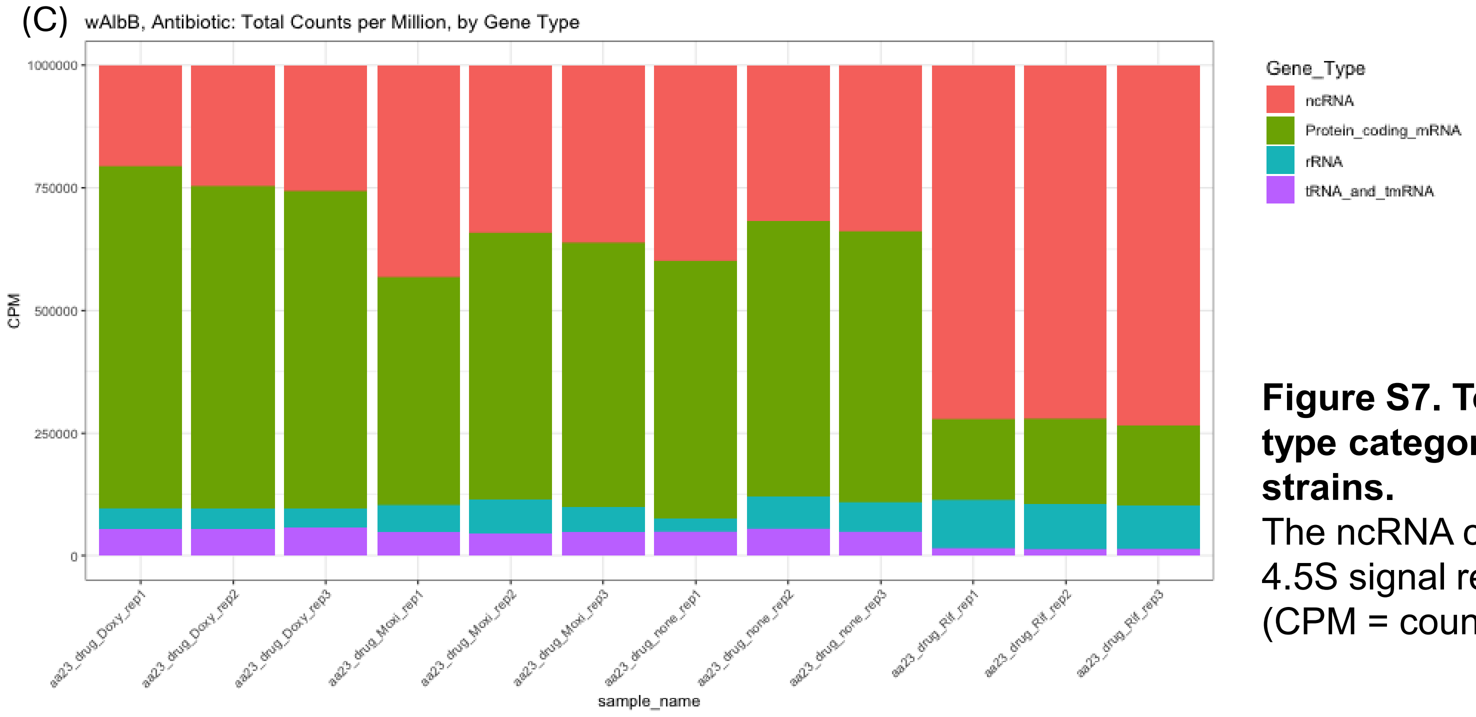

Figure S7. Total reads summed over the genes within various gene-type categories, under different treatments in the two *Wolbachia* strains.
The ncRNA category is comprised of three genes (i) 6S gene (*ssrS*) (ii) 4.5S signal recognition particle RNA (*ffs*), and (iii) RNase P RNA (*rnpB*). (CPM = counts per million reads mapping to *Wolbachia*)

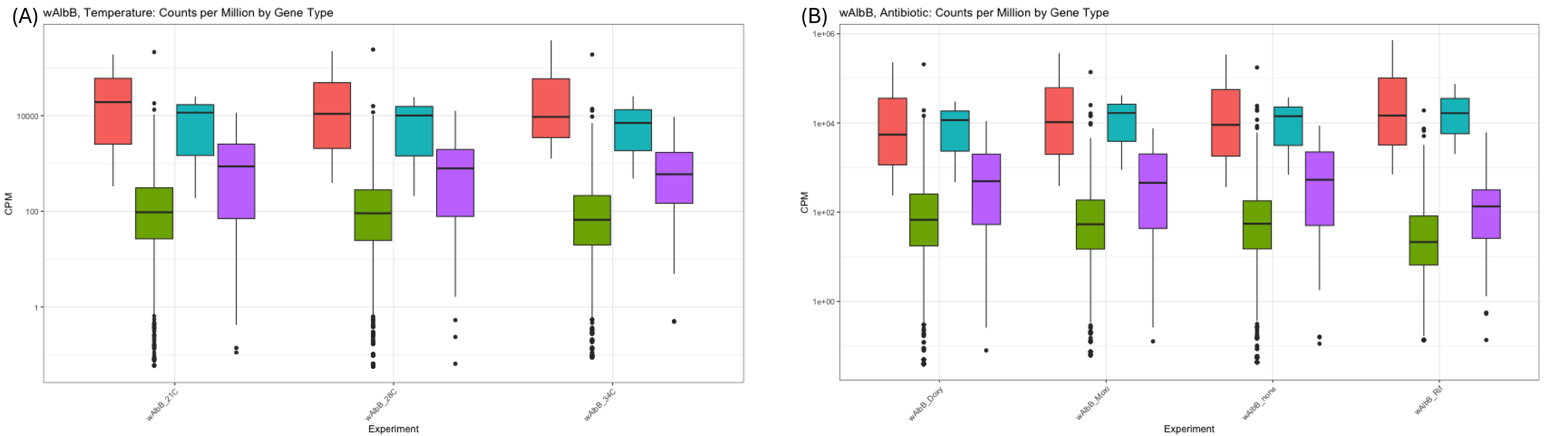

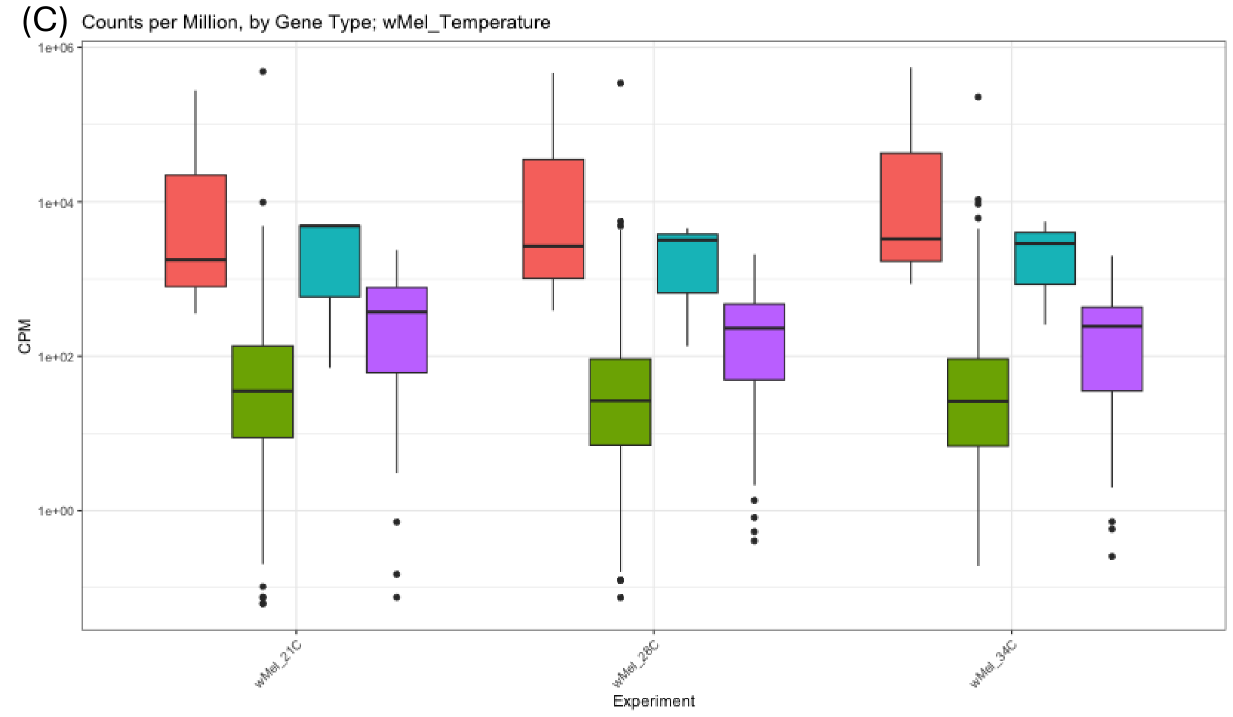

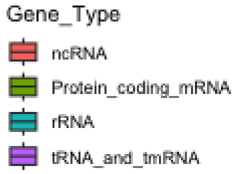

Figure S8. Distribution of read counts (log10 scale) per gene across various gene-types, under different treatments in the two *Wolbachia* strains.
The ncRNA category is comprised of three genes: (i) 6S RNA (*ssrS*) gene (ii) 4.5S signal recognition particle RNA (*ffs*), and (iii) RNase P RNA (*rnpB*). CPM = counts per million reads mapping to *Wolbachia*.

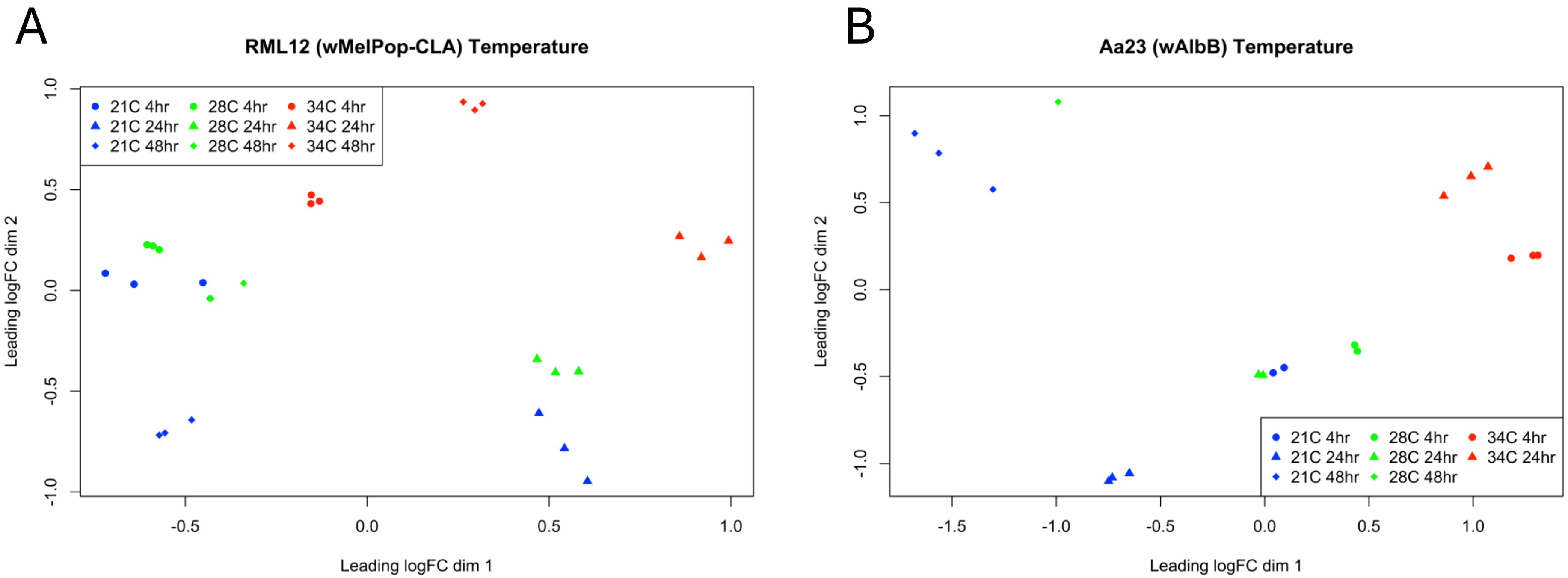

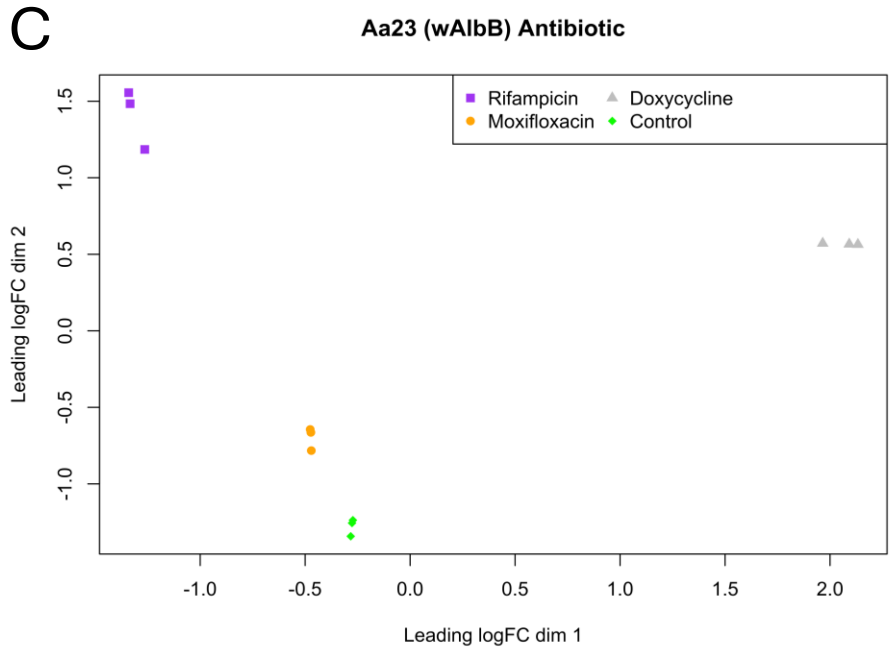

Figure S9. MDS plots of stress induced *w*MelPop-CLA and *w*AlbB samples. A) *w*MelPopCLA exposed to temperature stress, B) *w*AlbB exposed to temperature stress.

Figure S10. Comparison of fold-changes of TSS sites corresponding to single copy orthologs in *w*Mel and *w*AlbB.

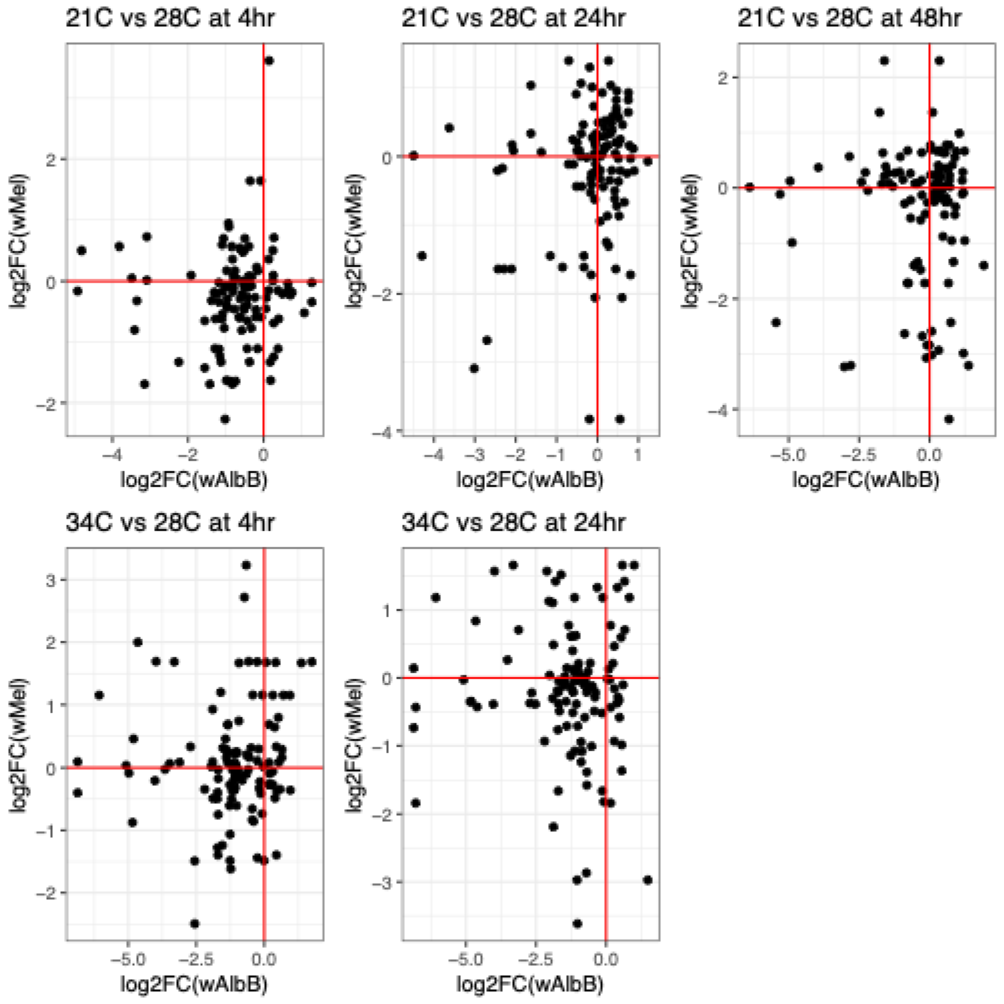

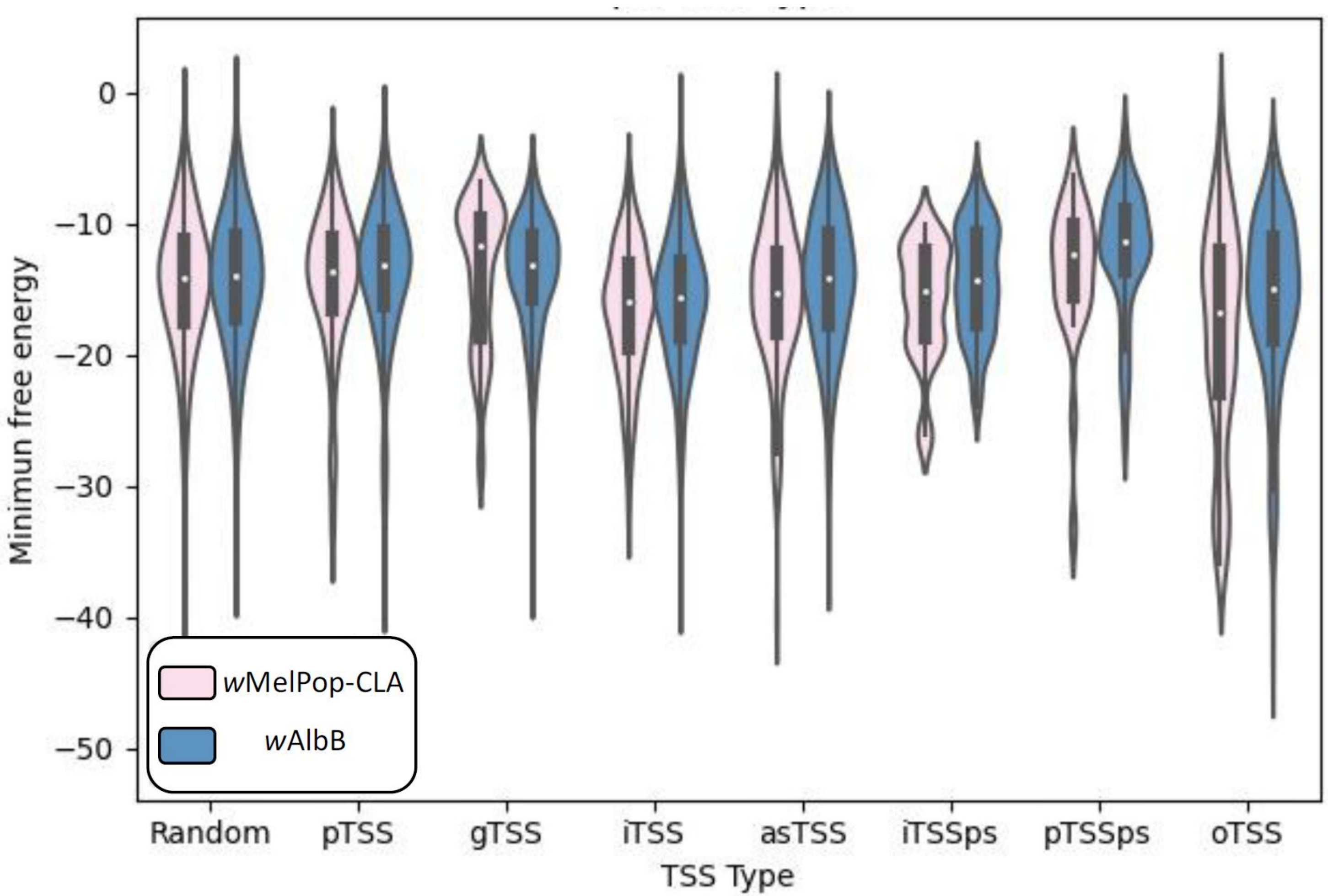

Figure S11. Violin plot of RNA folding minimum free energy distributions per TSS type for *w*AlbB and *w*MelPop-CLA. Central point represents median value.

Table S6. Summary of TSS RNA folding probability.

		Random	pTSS	gTSS	iTSS	asTSS	iTSSps	pTSSps	oTSS
w MelPop-CLA	Mean MFE	-14.68	-14.56	-13.73	-16.66	-15.52	-15.92	-13.95	-18.33
	n	2000	355	25	214	135	11	22	52
	signif		ns	ns	*	ns	ns	ns	*
w AlbB	Mean MFE	-14.23	-13.85	-13.94	-15.93	-14.45	-14.32	-12.08	-15.9
	n	2000	568	155	1229	785	86	54	199
	signif		ns	ns	*	ns	*	ns	*

*Independent Student t-test significance ($p < 0.05$) relative to random control.

Table S7. Summary of TSS-assisted operon prediction. Occurrence of predicted operons with number of associated genes for both *w*Mel and *w*AlbB genomes.

Genes in operon	wMel	wAlbB
28	1	
23		1
10	1	
9		1
8	1	
7	4	2
6	3	4
5	8	2
4	20	21
3	32	32
2	163	149
Total	233	212

Table S8. TSS types associated with TSS assisted operon prediction. Summary of operons with associated TSS types for both *w*Mel and *w*AlbB genomes.

	Operons	pTSS	gTSS	iTSS	asTSS
wMel	233	40	2	24	13
wAlbB	212	58	10	84	56
